# Contextual work design and employee innovative work behavior: When does autonomy matter?

**DOI:** 10.1371/journal.pone.0204089

**Published:** 2018-10-04

**Authors:** Christian P. Theurer, Andranik Tumasjan, Isabell M. Welpe

**Affiliations:** 1 Chair for Strategy and Organization, TUM School of Management, Technische Universität München, Munich, Bavaria, Germany; 2 Chair of Management and Digital Transformation, Gutenberg School of Management and Economics, Johannes Gutenberg University, Mainz, Germany; 3 Bavarian State Institute for Higher Education Research and Planning, Munich, Bavaria, Germany; University of Toronto, Rotman School, CANADA

## Abstract

In environments experiencing fast technological change in which innovative performance is expected, work design research has found that the degree of autonomy positively predicts behavioral and attitudinal work outcomes. Because extant work design research has tended to examine the direct and mediating effects of autonomy on work outcomes such as job satisfaction, examinations of more situational elements and the degree to which the organizational context strengthens or weakens this relationship has been neglected. This study, therefore, takes a context-contingent perspective to investigate the degree to which psychological climate dimensions such as supervisor support, organizational structure and organizational innovation moderate the effects of autonomy (work scheduling autonomy, work methods autonomy, decision-making autonomy) on employee perceived innovative work behavior (IWB). Using a conjoint experiment based on 9,440 assessments nested within 1,180 employees, it was found that all autonomy dimensions had a significant direct effect on employee perceived IWB. Contrary to the Hypotheses, the multi-level analysis did not reveal any moderating effect of the climate dimensions on the relationship between autonomy and employee IWB. This study provides a context-contingent view for the features of work design and gives a more detailed analysis of autonomy, which has previously been seen primarily as a unidimensional construct.

## Introduction

As the “increasing prevalence of technology” and the fast “changing nature of work” [1, 2] are impacting work processes and occupational structures in contemporary organizations (e.g., virtual teams), firms have been seeking to dynamically adapt work designs to best capitalize on their growing digitally aware workforce by leveraging their “digital fluency” [2–9]. At the same time, employees are seeking greater flexibility and self-determination and more individualized work schedules [10–13]. Therefore, organizations and especially organizations that have “loose alliances of [more] *autonomous* and multidisciplinary teams” are finding ways to foster greater innovation [14–16]. Especially fast-growing technology firms and today’s “digital star” firms put a high emphasis on employee autonomy to spur creativity [16, 17]. Netflix, for example, explicitly fosters a culture of “creativity and self-discipline, freedom and responsibility” as opposed to a “culture of process adherence” to attract and nourish innovative people and to sustain their success [16]. In doing so, Netflix has been highly successful and just achieved another record high with almost 110 million global subscribers in a business that has traditionally been with the large television networks and media conglomerates (status October 2017; [18]).

During the past few decades, research on work design has found that task/motivational, social and work context factors can significantly influence employee attitudinal, behavioral, cognitive, or organizational work outcomes [5, 19–21]. Of the task/motivational factors, *autonomy*, which is the individuals’ sovereignty when working [22], has been studied intensively and found to strongly predict positive attitudinal and subjective and objective behavioral work outcomes [19, 21, 23].

Even though workplace autonomy has had a long history in work design research, there has recently been renewed interest in this area (e.g., [12, 15, 24, 25]) primarily because of the increase in knowledge-based organizations in which enhanced employee discretion has been found to be an important predictor of innovative performance [13, 26]. Further, recent studies have found that autonomy can drive employee aspirations for power, in contrast to using power to gain influence over others [24].

Prior research has tended to focus on the fit (i.e., person-organization fit) between occupational demand and individual competence (e.g., [27, 28]). However, there has been much less focus on the extent to which organizational context inhibits or complements the evolution of a well-designed job or whether “certain job designs [i.e., degree of autonomy] may be more appropriate in certain contexts than in others” [1, 27, 29]. This paper proposes that only through a joint understanding of how autonomy and the broader organizational contexts or certain boundary conditions interact can a comprehensive understanding of employee attitudes, behaviors, and related work outcomes [27, 30] be achieved.

As the organizational context can either be treated as a main effect on work design features, or as a cross-level interaction/moderation effect with work design characteristics on work design outcomes [27, 31], in this paper, the focus is on the latter interpretation. Extant and emerging research taking a more contextual approach to work design has closely examined the social context of work design, such as the interpersonal interactions and relationships that are influenced by the work environment and the type of job. However, broader work context characteristics such as working conditions have not been widely examined [5, 7, 19]. To go some way to filling this gap, in this paper, the organizational context, or the “broader organizational environment in which employees work”; [27] is viewed as an important moderator in the relationship between work design and related outcomes [27, 29].

In particular, this paper examines how employee perceptions of the *psychological climate* dimensions (i.e., an individual’s perception of the work environment with regard to the broader organizational environmental dimensions) moderate the relationship between individual autonomy and employee perceived behavioral work outcomes such as innovative work behavior [32]. In line with these context-contingent perspectives, the specific climate conditions that moderate the influence of autonomy on employee perceived innovative work behavior (IWB) are examined. A conjoint analysis is conducted to test the different facets of autonomy and the impact they may have on the employee’s perception of their own innovative work behavior. Using a multi-level analysis, the moderating effects of selected psychological climate dimensions on this relationship are analyzed. As relatively few studies have addressed the effects of autonomy on entrepreneurial outcomes [33], this paper provides some guidance to employee support in the knowledge economy.

This paper contributes to the literature in at least two important ways. First, research on work design and specifically on autonomy is revisited from a context-contingent/boundary condition perspective. In doing, the recent calls are answered for enhanced research to consider the contextual features “that most powerfully constrain or enhance the emergence of well-designed jobs” within the broader organizational context [27, 29]. Therefore, a more comprehensive understanding of the supportive and inhibitive factors that affect autonomy relationships in task/motivational work design is illuminated.

Second, this study examines several dimensions of autonomy concurrently, thereby taking a different perspective than more recent studies (e.g. [1, 19]); for example, Hackman and Oldham [34] and most subsequent studies all viewed autonomy from a unidimensional perspective and tended to focus only on work scheduling autonomy [19]. Therefore, the study in this paper is a response to the need for research that recognizes that autonomy is multi-faceted and that these different facets can differentially impact work outcomes beyond just job satisfaction [21].

Because this investigation considers both work design-based autonomy dimensions and adjustable organizational-level properties or the psychological climate dimensions, it aims to improve the understanding of such reverse relationships; that is, how adjusted organizational boundaries impact work design. This additional insight is valuable as prior research has tended to (over)simplify reality by assuming this dimension to be fixed; however, new communication technology means that the organizational dimensions can also be modified [29].

The remainder of this paper is structured as follows. First, work design and contingency theory, the autonomy construct dimensions and the innovative work behavior concept are introduced and reviewed. Then, the contingent climate dimensions are introduced as the moderators of the relationship between the autonomy dimensions and employee perceived innovative work behavior after which the method and results are given. In the final sections, the discussion and conclusion are given.

## Theory and Hypotheses

Because of the need for enhanced context-contingent work design research that considers the interactions between work design features and the broader organizational context [27, 29, 30], work design theory [22, 35] and structural contingency theory are reviewed [36, 37] to derive a framework/model that can determine the impact that work design features, in this case autonomy, have on employee perceived innovative work behavior.

### Work design theory

Work design theory is based on the assumption that certain jobs, tasks or role characteristics as well as the broader social and contextual aspects of work engender psychological states such as intrinsic motivation that result in certain outcomes at the individual, group and organizational levels [21, 22]. This interrelationship also includes an implicit assumption that certain employee characteristics are present and that there is a match between the employee characteristics and organizational task requirements [21, 22].

Of all the identified motivational task-related work design features, *autonomy* or “the degree to which the job provides substantial freedom, independence and discretion to the individual” [22] has been found to strongly affect both subjective and objective employee performance (e.g., creativity) and attitudinal outcomes such as commitment and job satisfaction [19, 21, 38]. For knowledge workers, in particular, autonomy has been found to be an important, essential aspect of their performance [39]. Further, it has been shown that *proximal* work environment characteristics such as job complexity and autonomy are more important than *distal* characteristics such as organizational policies in predicting employee creativity [40]. Therefore, this paper focuses on autonomy as a work design feature and as a predictor of employee perceived innovative work behavior.

### Contingency theory

**Fig 1:**
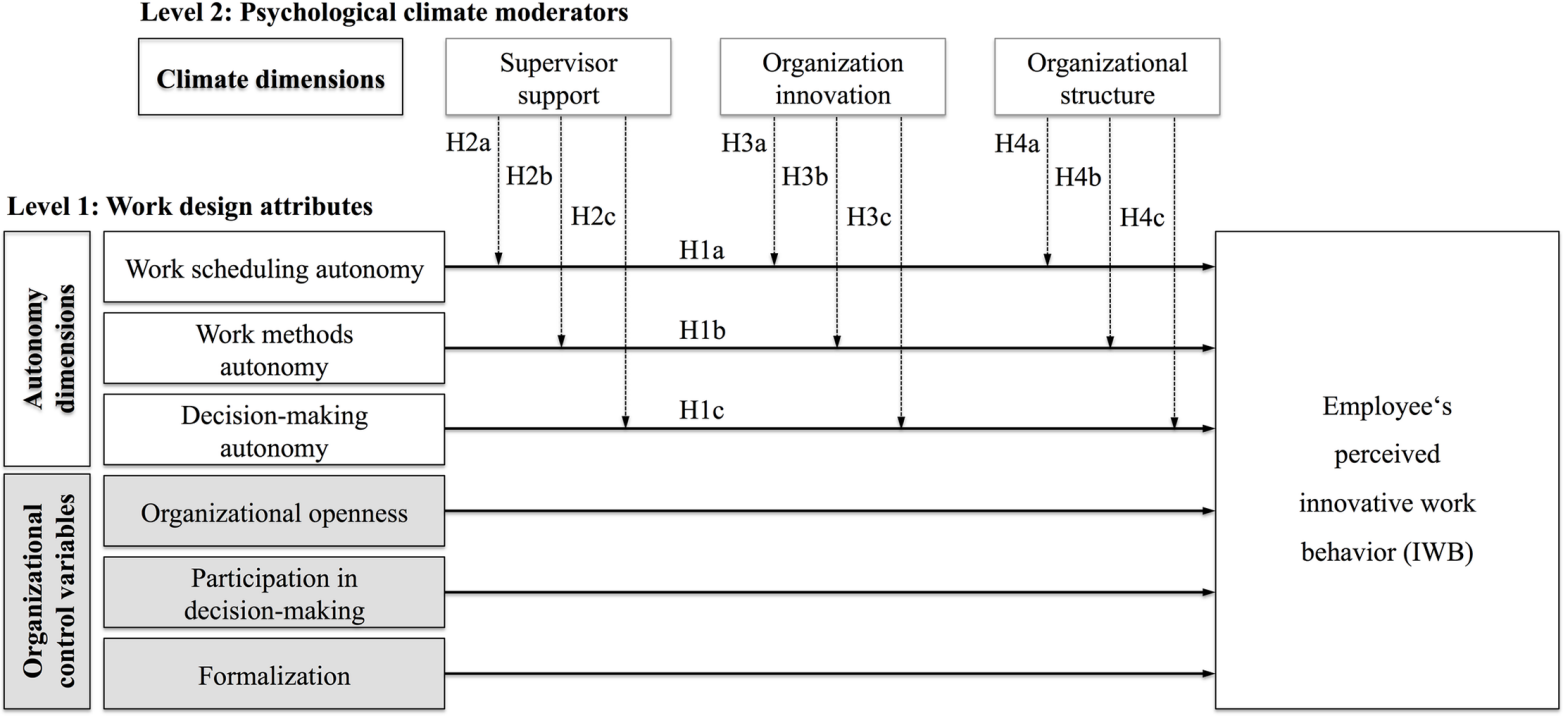
Conceptual model showing the direct effect of work design features on employees’ perceived IWB and the moderating role of psychological climate dimensions.

Structural contingency theory assumes that optimal organizational set-ups are contingent on the specific external and internal circumstances that an organization or individual faces, and that there is a fit between set-ups and these circumstances [41, 42]. This perspective has been examined in a wide variety of contexts by, for example, addressing national contexts and cultures (e.g., [43]), leadership (e.g., [44]), technology (e.g., [45]), or individual traits (e.g., [46]) as contingency factors. In the model (see Fig 1), these current views are extended by considering how the autonomy dimensions are related to employee perceptions of their own work climate.

### Autonomy and work design-related outcomes

Traditionally, autonomy has been considered a job/task characteristic of work design and has been based on an intrinsic motivational paradigm in which several personal and work outcomes such as innovative performance are rooted [21, 47, 48]. Autonomy in an organizational work context has empirically been associated to individuals or groups and can be practiced in higher-level, lower-level and knowledge worker contexts [49, 50].

Autonomy has been found to positively predict various behavioral outcomes such as objective and subjective employee performance and absenteeism, attitudinal individual and group level outcomes such as job satisfaction, job involvement and organizational commitment, and organizational outcomes such as customer satisfaction [5, 19, 51]. Autonomy is considered of particular value in so-called “adhocracy cultures” in which “an idealistic and novel vision [that] induces members to be creative and take risks” results in enhanced risk-taking and greater innovative adaptability [52]. On the downside, however, it has also been shown that high levels of autonomy and low levels of monitoring can result in lower team performance than high levels of autonomy and high levels of monitoring [53], implying that putting too much trust in an autonomous team can also be detrimental.

### Dimensions of autonomy

Autonomy is not a one-dimensional construct but has several dimensions [19]. In contrast to the views taken in the 1970s and 1980s, autonomy is now understood to be a multi-faceted construct that encompasses more than just strategic autonomy and control over work goals [33]. Today, the dimensions have been extended to *work scheduling* autonomy, *work methods* autonomy, and *decision-making* autonomy, each of which differentially predict work outcomes [19, 33, 35, 54, 55]. Work scheduling autonomy at either the individual or group level refers to having control over the timing and scheduling of work, work methods autonomy refers to having control over the procedures and methods used to do the work and decision-making autonomy refers to having the freedom to make work-related decisions [19]. While these dimensions are inherently related, each one has distinct predictive abilities [19, 21].

### Innovative work behavior

Innovation or creativity as an outcome of work design characteristics have rarely been the central focus of research into work design outcomes [13, 19] and have generally been regarded as “expanded” or “distal” outcomes [5, 13]. However, creativity and innovation are vital for organizational effectiveness [56, 57] as organizational success is often dependent on employees who exceed “standard work behaviors” by being innovative rather than merely fulfilling their formal work requirements as stated in the job description [58].

An employee’s innovative work behavior is dependent on a combination of three different behavioral tasks: the *generation of ideas*, the *promotion of ideas*, and the *realization of ideas* [58–60]. While innovative behavior involves both the promotion and realization/implementation of ideas, the creativity concept has been seen to be only involved in idea generation [58, 61].

The antecedents of employee creativity and the elements of innovative work behavior (e.g., [40, 62]) have been widely examined. For example, Scott and Bruce [62] studied the influence of leadership, work group relations and individual attributes on innovation in the workplace and found that the supervisor-subordinate relationship, supervisor role expectations, and employee systematic individual problem-solving styles predicted high levels of innovative behavior. Similarly, Yuan and Woodman [56] evaluated the influence of the expected outcomes of innovative behavior, such as expected performance outcomes or expected image gains, on employee innovation and found that both performance expectations and image consequences had a significant impact on employee innovation.

### Autonomy and innovative work behavior

From a work design motivational perspective [22], based on an index of job characteristic dimensions including job autonomy, it was found that job complexity was positively related to supervisor-rated employee creativity and performance [57]. Several studies have also found a positive relationship between work design features such as autonomy and creativity and innovation at work (e.g., [48, 63–65]). For example, Dul and Ceylan [66] investigated the influence of a creativity-supporting work environment (e.g., challenging job, teamwork, job autonomy) on firms’ new product introduction to the market and showed that the more a firm’s overall work environment supports creativity, the higher the firm’s percentage of sales from new products. In a similar vein Ramamoorthy, Flood [48] directly and indirectly tested the influence of job autonomy on innovative work behaviors when mediated through an obligation to innovate and found that job autonomy had a direct positive effect on innovative work behaviors. Autonomy has further been found to be an influential moderator in the relationship between leadership styles and relationships and creativity at work (e.g., [67, 68]).

The Hypotheses are first introduced in relation to the main effects of the autonomy dimensions, then, the influence of the climate dimensions as moderators on the relationship between autonomy and employee perceived innovative work behavior is examined.

#### Work scheduling autonomy and innovative work behavior

Originally from a manufacturing context, work scheduling autonomy has been defined as the “extent to which workers feel they can control the sequencing/timing of their work activities” [54]. Employees that are not tied to any specific schedules or timing can, therefore, freely choose when and in which order they want to pursue certain tasks, and thus exert the related behaviors [55].

In comparison to standard tasks in positions with more “discrete, sequential stages,” innovation and thus innovative behavior is characterized by discontinuous, intermittent, alternating activities and behavior [62, 69]. Therefore, it is assumed that when employees are able to freely choose when and in what order they work on different tasks, their intrinsic motivation is activated, which positively impacts innovative work behavior in terms of idea generation, idea promotion, and idea implementation, implying the following relationship between work scheduling autonomy and employee innovative work behavior.

**Hypothesis 1a.** Higher levels of *work scheduling autonomy* are associated with higher levels of *perceived innovative work behavior*.

#### Work methods autonomy and innovative work behavior

Work methods autonomy has been defined as the “degree of discretion/choice individuals have regarding the procedures (methods) they utilize at work” [54]. As innovative behavior at work reflects a “complex behavior” comprised of “interrelated sets of behavioral activities” such as problem recognition, idea generation, idea promotion, and idea realization [70, 71], it appears critical that employees are able to freely choose *how* to approach these stages. This type of autonomy has been found to be particularly important during the initial idea generation phase [71].

There are many techniques that can be used for new idea generation such as brainstorming, mind mapping and morphological analysis [72]. Therefore, limitations defined by an organization toward a certain approach or having a pre-specified selection or set of certain methods and instructions might negatively impact employee creativity and idea generation [71, 72]. Employees might also feel limited in the options they can choose to address certain problems, reducing their motivation to be innovative. It is therefore assumed that there is the following relationship between work-methods autonomy and innovative work behavior.

**Hypothesis 1b.** Higher levels of *work-methods autonomy* are associated with higher levels of *perceived innovative work behavior*.

#### Decision-making autonomy and innovative work behavior

The third autonomy dimension is related to the freedom to make decisions about work [19, 73]. As the two core phases of the innovation process are initiated through idea generation and implemented through idea fulfillment, many major and minor decisions need to be made along the way such as the decision to innovate, the decision to proceed with a certain idea and the decision to implement [74, 75]). Therefore, the interrelated stages in innovative work behavior (see [70]) require ongoing decision-making within the stages and between the stages; that is, from idea generation to idea implementation [74].

Low decision-making authority along this iterative process could result in the constant need to seek approval from decision-makers, thus constraining motivation and related behaviors [51]. Therefore, it is assumed that when employees are able to freely make decisions about the direction in which to proceed rather than having to seek supervisor approval or abide by restrictions, there is a positive influence on their innovative work behavior and performance, which implies the following interaction between decision-making autonomy and innovative work behavior.

**Hypothesis 1c.** Higher levels of *decision-making autonomy* are associated with higher levels of *perceived innovative work behavior*.

Of these three autonomy dimensions, meta-analysis extant findings have found that work scheduling autonomy, in comparison to work methods autonomy and decision-making autonomy, has relatively little impact on job satisfaction, and that the different autonomy dimensions have distinctive predictive effects [19]. While most studies have focused on job satisfaction, there is also evidence of similar differential autonomy effects for employee innovative work behavior.Axtell, Holmann [50], for example, found that different forms of autonomy on the shop floor such as control over machine maintenance vs. control over working methods had differential effects on idea generation and creativity. Translated to the context of this study, it is therefore assumed that the different autonomy dimensions have distinctive predictive effects on innovative work behavior, implying the following:

**Hypothesis 1d.** *Work methods autonomy* and *decision-making autonomy* have a significantly larger effect on perceived employee innovative behavior than *work scheduling autonomy*.

### Organizational context as a moderator of work design relationships

Prior work design research has highlighted the importance of contextual features in constraining or fostering the development of well-designed jobs [27, 29]. Context has two analysis levels: the global *omnibus* context, within which is nested the specific *discrete* context, which involves the variables that determine certain attitudes and behaviors [31]. From a work design and contingency perspective, context is important because employees seek to attain correspondence or fit with the broader context that “reinforces or rewards different individual needs and behaviors” [27]. Work designs related to the previously outlined autonomy dimensions, therefore, allow employees to attain correspondence, and can therefore create positive attitudinal and behavioral work outcomes [27]. Context can influence employee intrinsic motivation which, in turn, can have an effect on employee creativity [57, 64].

There have been some studies focused on the broader organizational and occupational context [27, 76]. Specifically, it has been suggested that organizational climate or the “shared perceptions regarding formal and informal organizational policies, practices, and procedures” [27] can impact work design characteristics “by making specific features more salient” and by “shaping the meaning of work design characteristics in specific ways” [27]. Likewise, on the *individual* level, the *psychological climate* describes an “*employee’s perception* of the work environment” along the different organizational dimensions; for example, between the *task climate* and the *relational climate* [77, 78]. While the construct of organizational climate describes perceptions of organizational practices on a shared level (e.g., work group, department within an organization), psychological climate considers individual perceptions of the work environment. In this study, the focus is on the individual level and thus on specific psychological climate dimensions of the organizational context.

### Psychological climate

Extant climate studies have demonstrated that the perceptions of the different psychological climate dimensions link organizational climate characteristics and employee attitudinal and behavioral outcomes such as motivation, job satisfaction, psychological well-being and performance [32, 77, 79]. Job satisfaction and motivation have consistently been identified as mediators between climate dimensions and work outcomes in terms of performance [32, 77, 79]. Of the many climate categories, *affective* [28], *work group and social environment* [80] and *relational or task climate characteristics* [77] have been found to have the strongest relationships with work outcomes [32, 77, 79].

On the basis of selected psychological climate dimensions, a theoretical model is developed to examine the moderating role of the climate dimensions on the relationship between the autonomy facets and employee perceived innovative work behavior. For the classification and selection of the relevant climate dimensions and the taxonomy, as it has been successfully applied in a range of climate studies (e.g., [79]), Ostroff’s [28] psychological involvement framework was adopted, which is made up of the *affective*, *cognitive*, and *instrumental* states in the workplace. In this paper, one affective (supervisor support), one cognitive (organization innovation), and one instrumental (organizational structure) climate dimensions were selected to examine the influence of employee autonomy evaluations on employee IWB.

#### The moderating influence of supervisor support

The *affective* climate dimension addresses employee interpersonal and social relations at work including cooperation, participation, warmth and social rewards [28, 79]. *Supervisor support* as a measure of cooperation is characterized through employee “support and understanding from their immediate supervisor” and the “extent to which the supervisor […] encourages the development of close, mutually satisfying relationships within the group” [77, 81]. The importance of supervisor support has been highlighted in previous work, in which it was found that good leader-member exchanges (LMX) are directly and positively related to work outcomes and innovative behavior (e.g., [57, 62, 67, 68, 82]).

The focus of this section, however, is the moderating impact of supervisor support on the relationship between the autonomy dimensions and employee IWB. It is expected that (high) perceived supervisor support, which includes supervisor empathy, confidence, guidance and a good understanding of the people working for them, increases the effect of autonomy on employee perceived IWB [67, 81]. In short, high supervisor support results in good leader-member relationships, which are related to high(er) levels of trust between the employee and the supervisor [57]. Employees that experience a superior LMX relationship often show reciprocal behavior that is reflected in greater discretionary work processes within the supervisor-subordinate relationship [83]. This implies that from both a leader and employee point of view, discretionary behavior is inherent in or triggered by work climates that have good leader-member cooperation.

Therefore, when employees perceive high levels of supervisor support, this strong, mutual basis of trust inherently ‘granted’ by the good LMX relationship encourages self-efficacy, flexible role orientation and proactive behavior, making employees feel more comfortable with greater autonomy [84]. Hence, as employees need to spend less time on establishing and maintaining their relationship with their supervisor for work success [85], they can take advantage of the high levels of autonomy to indulge in more innovative behavior. In contrast, employees who have low supervisor support levels and thus lack guidance, trust and self-efficacy might not (yet) feel comfortable being given greater autonomy as they might first want to establish a closer relationship with their supervisor to develop the trust needed for autonomy to be activated, implying the following:

**Hypothesis 2.** The effect of a) work scheduling autonomy, b) work methods autonomy, and c) decision-making autonomy on perceived innovative work behavior (IWB) is moderated by perceived *supervisor support*, such that under higher levels of perceived *supervisor support*, the importance of a) work scheduling autonomy, b) work methods autonomy, and c) decision-making autonomy is higher.

#### The moderating influence of organizational innovation

The second climate dimension, the *cognitive* facet, is related to personal development and employee involvement in work activities. Cognitive climate includes innovation, growth and intrinsic award dimensions [28, 79], all of which have been found to have positive effects on innovative work outcome orientation (e.g., [77]). O*rganizational innovation*, defined as the “perceived emphasis on innovation and creativity in work; [the] acceptance of change” [77], therefore, has been chosen in this paper as representative of the cognitive climate dimension.

It is expected that perceived organizational innovation has a positively moderating role on the autonomy-IWB relationship. An innovative organizational climate is characterized by the encouragement for, support for and the rapid, flexible adoption of new ideas and a culture that encourages employees to search for new problem-solving techniques and approaches [81, 86]. Employees who perceive they work in environments driven by an innovative organizational focus are therefore constantly surrounded, and potentially pressurized, by a mindset and thus target setting for creativity and innovation performance [86, 87]. As discussed, autonomy is necessary to promote employee creativity, and is therefore essential for success in an innovative organizational context (e.g., [48, 57, 64]).

When employees work in a highly innovative organizational climate, the need (and pressure) to innovate is more likely to be more important for the organization’s success; therefore, the positive effect of (more) discretion on employee innovative behavior might also be higher. Organizations that have low innovation or less of a need to innovate, on the other hand, may divert employee focus to other organizational goals and restrain employee autonomy, implying the following:

**Hypothesis 3.** The effect of a) work scheduling autonomy, b) work methods autonomy, and c) decision-making autonomy on perceived innovative work behavior (IWB) is moderated by perceived *organizational innovation*, such that under higher levels of perceived *organizational innovation*, the importance of a) work scheduling autonomy, b) work methods autonomy, and c) decision-making autonomy is higher.

#### The moderating influence of organizational structure

The third climate dimension, the *instrumental* facet, is concerned with work processes and task involvement and is represented by constructs such as extrinsic rewards, structure, hierarchy, and achievement [28, 79]. In line with an innovative organizational climate, as *organizational structure* has been one of the core dimensions and most commonly measured factors in the psychological climate [77], in this paper structure is chosen to be representative of the instrumental climate dimension. Organizational structure is generally defined as the “perception of formality and constraint in the organization, orderly environment; emphasis on rules, regulations, and procedures” [77]. Organizational structure is usually characterized by rules, pre-specified procedures, processes, or technicalities and an enhanced focus on an adherence to guidelines and instructions [81]. It is important to note that structure is distinct from centralization and hierarchy, which refers to the decision-making authority locus [88, 89].

Organizational structure also provides “speed, efficiency, and reliable and consistent performance” [88, 90] and has also been found to encourage (intrinsic) motivation, flexibility, and innovation [88, 91–94]. However, in this paper, the view that organizational structure diminishes the effect of autonomy on employee perceived IWB is taken. Organizations with definitive structures have many (pre)defined processes, rules and regulations in place which can stifle innovation and innovative success (e.g., [95]).

Creativity and innovation require a degree of flexibility and freedom so as to motivate employees [90]; however, if scheduling and decision making are bound with a definitive structure, employees have less experience with outcomes, success rates, and organizational consequences because they have less autonomy and therefore show less innovation than in more discrete working environments and might even face negative organizational consequences by not adhering to the formal rules. In these organizations, therefore, conformity with organizational guidelines is valued more highly than personal satisfaction and motivation (i.e., extrinsic vs. intrinsic motivation; [87]).

When there are high perceived structural levels, employees are more likely to succeed if they follow the given processes as in highly structured organizations, employees’ personal motivation and satisfaction is subordinate. Any deviance from these given rules and regulations such as enhanced autonomy and greater freedom would therefore endanger this success. Less structured organizations, however, provide greater freedom and encourage/allow higher levels of autonomy when seeking to achieve certain outcomes in line with organizational regulations, implying the following:

**Hypothesis 4.** The effect of a) work scheduling autonomy, b) work methods autonomy, and c) decision-making autonomy on perceived innovative work behavior (IWB) is moderated by perceived *organizational structure*, such that under higher levels of perceived *organizational structure*, the importance of a) work scheduling autonomy, b) work methods autonomy, and c) decision-making autonomy is lower.

## Data and method

### Research instruments and experimental design

To examine the organizational conditions under which employees perceive innovative behavior, a conjoint analysis was conducted followed by a post-experiment questionnaire on the participant backgrounds and the characteristics of their organizations. The conjoint experiment was conducted to analyze the direct effects between dimensions of autonomy and employees’ perceived innovative work behavior. The post-experiment questionnaire was used to capture the respondents’ demographic background, as well as to assess the moderator variables (i.e., supervisor support, organizational innovation, organizational structure). The translated questionnaire and conjoint experiment profile is provided in the S1 Appendix in the Supporting Information section.

Conjoint analyses have been frequently conducted in various disciplines such as marketing (e.g., [96, 97]), entrepreneurship (e.g., [98, 99]) and human resource management (e.g., [100, 101]) to evaluate complex decision-making processes [102]. For example, Brundin, Patzelt [99] investigated in a conjoint experiment the impact of managers’ emotional displays (e.g., confidence, satisfaction) and their impact on employees’ willingness to act entrepreneurially. Employees had to make a series of judgments based on developed profiles that described hypothetical decision situations comparable to the ones in this study. In another study Baum and Kabst [100], for example, examined the importance of different organizational characteristics and their impact on employees’ job choice through conjoint analysis.

Compared to post-hoc methodologies such as surveys or interviews, conjoint analyses can overcome certain biases such as self-report bias when respondents answer in a way that “makes them look as good as possible” with regard to socially desirable behaviors [103], or retrospective/recall bias/telescoping where respondents show differences when recalling information about a past experience or event by overstating recent events and understating more distant events [99, 104, 105]. Furthermore, when using for example rating scales to examine the importance of certain organizational characteristics respondents tend to rate every item as important and it is difficult to collect contingent decision data in a specific (hypothetical) situation [106]. Conjoint analysis, however, is well suited to investigating “interactions among decision criteria” and make a real-time decision considering different factors simultaneously [106]. Using conjoint analysis therefore avoids certain limitations related to the use of post-hoc methods and is therefore well suited to study the impact of different dimensions of organizational autonomy on employees’ perceived innovative work behavior.

In conjoint experiments, participants are generally required to assess a series of different, hypothetical profile sets which have a certain combination of attributes or cues that assume certain levels or values for each profile [107]. Participants evaluate each profile set and make judgments in relation to a certain outcome variable, such as innovative behavior at work [107]. These series of judgments made by each participant allows for an analysis of the underlying structure of the decision making to deduce the relative importance of each attribute and to analyze how the contingency relationships are processed as the participants prioritize the attributes [108, 109]. The underlying structure of the decisions were analyzed using hierarchical linear modeling (HLM) to account for the decisions nested in the individual participants [110].

A metric conjoint experiment with an orthogonal design which had zero correlation between all possible attribute level combinations was designed for this experiment [102]. The experiment had six attributes, with three being related to the Hypotheses and three being used as controls for comparative reasons (see next section). Each attribute varied between two possible, opposing conditions (high and low), which resulted in a possible set of 64 (2^6^) profile combinations. By applying a fractional factorial design [111] the total number of possible combinations of attributes and profiles was reduced to eight, which were then fully replicated using test-retest correlation to account for reliability [102].

**Fig 2:**
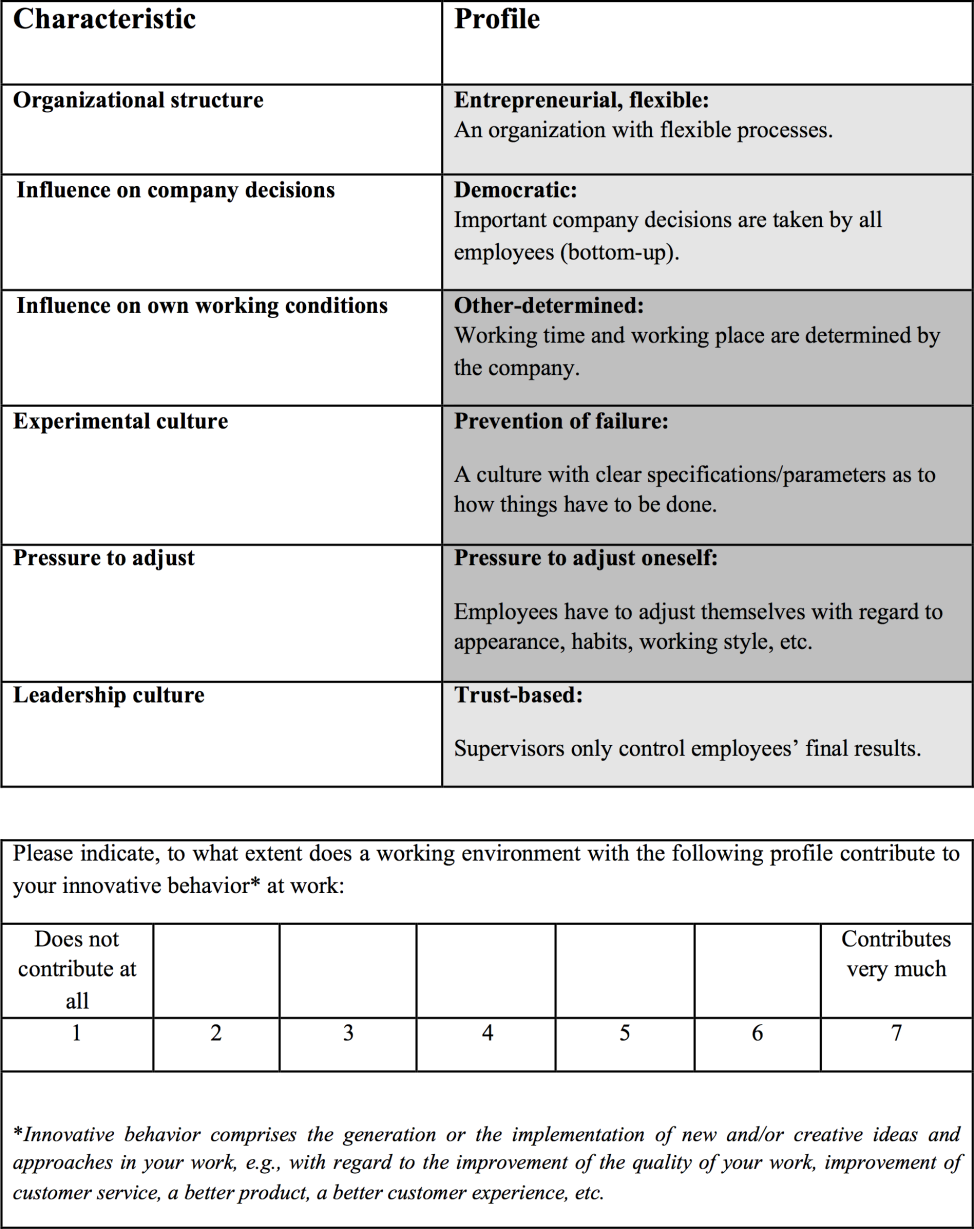
Sample conjoint profile as used in the survey (English translation).

Therefore, there were 16 profiles for each participant (2 x 8 sets) rather than the theoretical 128 profiles. Prior to the determination of the 16 profiles, each participant was provided with a sample profile (see Fig 2) so as to become familiar with the structure of the succeeding profile sets. To control for ordering effects, participants were randomly assigned to different versions of the profile sets. Specifically, two versions were created to control for the *attribute order* of appearance *within* a decision profile, and two versions were created to account for the *profile order across* all decision profiles [109].

### Sample

The sample was recruited through an online panel provider that distributed a web link to the survey. Participants were incentivized through the panel provider and received a fixed remuneration for their participation in the study. The remuneration was only paid when the study was successfully completed. Participants that did not finish the survey or provided invalid answers were not remunerated in the end. All data were collected and analyzed fully anonymously and did not allow drawing any personalized inferences about participants. The authors did not seek approval by an institutional review board (ethics committee) because identifying information about survey respondents was not collected, used or reported at any stage of the study. The survey did neither collect any sensitive information (e.g., religion, nationality, state of health) nor personal information (e.g., name, (e-mail) address, phone number) of the participants. At our department there is no institutional review board and there exists no need to seek approval from such a committee for survey-based or experimental research in the field of social sciences, and more specifically organizational behavior and applied psychology.

The approach followed the department’s standard research procedure in which all respondents are clearly instructed at the beginning of the study that their responses will be used for a research project or series of studies at the institution and that all information will be treated fully confidentially. Furthermore, the panel provider remunerated participation in the study and participation therefore assumed an agreement to use the survey responses for research purposes and further publication of the results. Through accepting the terms and conditions as well as the privacy policy of the panel provider, the participants provided their general consent to use, store and process their data for relevant purposes (e.g., scientific research). As a quality criterion the panel provider restricts participation in their studies to an overall maximum of 12 studies per year and participant and not more than two studies on average per month and participant.

The online panel provider pre-selected candidates based on defined company size quotas: 1–10 employees: 15%; 11–499 employees: 35%; 500–5,000 employees: 35%; >5,000 employees: 15%; and employee professional qualifications: university degree: 40%; vocational training: 60%. Soft quotas were agreed for gender: 50% female, 50% male; and age: 18–24 years: 15%; 25–34 years: 19%; 35–44 years: 24%; 45–54 years: 24%; 55–65 years: 18%. At the beginning of the survey, participants were asked about their highest educational degree (“What is your highest professional qualification?”), employment status (“Are you currently employed full-time or part-time?”) and age (“Please indicate your age”) to ensure that data was obtained only from people currently employed in Germany who had completed a professional qualification and were between 18 to 65 years old. People who did not match the age criteria, were not currently employed part-time or full-time or did not have a professional qualification were not able to proceed to the main part of the study.

A pre-test was conducted on 16 participants to gain useful insights and suggestions for improvements with regards to timing, the clarity of the phrasing and definitions and the reliability of the conjoint decisions. For the main study, 2,550 employed individuals from across Germany completed the survey in February 2016. Among all participants the average duration of the survey was 25.1 min (SD = 15.7) including the pre-screening questions, the conjoint experiment and the post-experiment questionnaire on the measures for the chosen psychological climate dimensions. Certain checks were then applied to ensure sufficiently high reliability to guarantee the quality of the dataset. Specifically, for each participant, the correlations between the 8 original decision profiles and the 8 replicated profiles were computed. In line with Shepherd, Patzelt [102] only participants with a correlation of at least .3 between the original set of profiles and the replicated profile set were included. Unreliable answers were also rejected, with the final analysis being conducted on a sample of N = 1,180 with a mean test-retest correlation of .65 across the two profile sets, which was in line with similar studies [102, 108]. Among the final sample of 1,180 respondents the average duration of the conjoint analysis and the post-experiment questionnaire was 27.3 min (SD = 16.1).

The final sample consisted of 82.5% full-time employees, with the remainder being part-time employees working an average of 51.6% (SD = 19.1) of full-time work. Just over half (50.3%) the respondents were female and the average age across the participants was 42.5 years (SD = 12.1), with 53.8% having completed a university degree and the remainder having completed an apprenticeship/vocational training in areas such as insurance/retailing incl. banking, insurance, hospitality, artisanry and public administration. The mean number of years of professional experience years was 20.7 years (SD = 13.0): 16.2% had professional experience of up to five years and 24.7% had been working for 31 or more years. Only 5% has been employed for 31 or more years with their *current* company, whereas 39.3% had been working for up to five years for their current company. The average number of years of being employed in their *current* company was 10.8 years (SD = 9.8) and the majority of participants worked for smaller companies up to 499 employees (52.3%), 31.9% worked for companies between 500 and 5,000 employees, and 15.8% worked for companies with more than 5,000 employees.

Participants worked in a range of industries: services (40.0%), manufacturing (16.4%), retail and wholesale (10.8%), public administration (9.8%), and others including transport, communication, energy (9.0%), or financial, insurance, property/real estate (8.0%). Participants worked in: (general) management and administration (22.3%), marketing/sales/communication (11.5%), fabrication/manufacturing (9.3%), information technology (8.5%), procurement and logistics (8.1%), research and development (8%), finance (5.2%), and human resources (4.7%), with 30.3% of these having staff responsibilities, 17.9% having budget responsibilities and 7.9% being owners/shareholders of their companies.

### Variables and measures

#### Assessment of employee perceived innovative work behavior. Dependent variable

Each conjoint profile had six attributes with each attribute having one of two opposing predetermined levels. The different attribute levels were highlighted with different colors. For each decision profile, the participants’ perceived *innovative behavior* was analyzed (i.e., “To what extent does a working environment with the following profile contribute to your innovative behavior at work?”) as in Scott and Bruce [62]. In line with Hurt, Joseph [112], innovative behavior was further specified and defined in each profile as “*innovative behavior comprises the generation or the implementation of new and/or creative ideas and approaches in your work*, *e*.*g*., *with regard to the improvement of the quality of your work*, *improvement of customer service*, *a better product*, *a better customer experience*, *etc*.”. Answers were given on a seven-point Likert-type scale ranging from “1 = does not contribute at all” to “7 = contributes very much”. A sample profile is provided in Fig 2.

#### Level 1. Decision attributes

Overall, for each conjoint profile, the effects of the six categorical attributes on the dependent variable were examined, three of which were related to the Hypotheses and the three autonomy dimensions: work scheduling autonomy, work methods autonomy, decision-making autonomy; and the additional three acting as control variables for comparative reasons which addressed additional organizational features that could also possibly impact the decision-making process: organizational openness, participation in decision-making, and formalization (e.g., [113]). Participants were asked to assume that all variables not specified in the profiles should be assumed to be constant across all decision situations. Table 1 illustrates and summarizes the attributes and their respective conditions.

**Table 1:** Organizational attributes and the different states, as used in the conjoint analysis.

Attribute	Description
**Influence on own working conditions** *(Variable*: *Work scheduling autonomy)*	**Self-determined**: Working time and working place can be freely chosen by the employee.
	**Other-determined**: Working time and working place are determined by the company
**Experimental culture** *(Variable*: *Work methods autonomy)*	**Learning from mistakes**: A culture that allows employees to try out new things.
	**Prevention of failure**: A culture with clear specifications/parameters as to how things have to be done.
**Leadership culture** *(Variable*: *Decision-making autonomy)*	**Trust-based**: Supervisors only control employees’ final results.
	**Control-based**: Supervisors continually control employees’ working progress.
**Pressure to adjust** *(Variable*: *Organizational openness)*	**Freedom to be oneself**: Employees do not need to adjust themselves with regard to appearance, habits, working style, etc.
	**Pressure to adjust oneself**: Employees have to adjust themselves with regard to appearance, habits, working style, etc.
**Influence on company decisions** *(Variable*: *Participation in decision- making)*	**Democratic**: Important company decisions are taken by all employees (bottom-up).
	**Hierarchical**: Important company decisions are exclusively taken by the management team (top-down).
**Organizational structure** *(Variable*: *Formalization)*	**Entrepreneurial, flexible**: An organization with flexible processes.
	**Bureaucratic, standardized**: An organization with standardized processes.

As *work scheduling autonomy* is based on employee control over the scheduling of their work [54, 55, 114], employees therefore have discretion in terms of their working time and place. Therefore, work scheduling autonomy was denoted as having an “*influence on own working conditions*”. This was further divided into two levels: “s*elf-determined*: *working time and working place can be freely chosen by the employee”* [high] and “*other-determined*: *working time and working place are determined by the company”* [low].

As *work methods autonomy* refers to the choice or discretion that an employee has over the procedures they utilize in their work [54] and implies that employees are free to choose *how* things are to be done at work, employees are therefore free to experiment and adjust their methods to attain their goals. Pre-specified methods in highly process-driven organizations are usually applied to standardize and to mitigate risks as the outcomes are more predictable [92, 115]. Such discretion can have multiple origins within an organization such as from within the organizational structures, or as part of the systems and processes. Discretion, therefore is expressed/manifested in an organization’s culture through the “shared values and norms that guide employees’ interactions with peers, management, or clients” [81, 116]. To account for the possible multiple origins, work methods autonomy was therefore operationalized as “*experimental culture*” which was divided into two levels: “*learning from mistakes*: *a culture that allows people to try out new things*” [high] and “*prevention of failure*: *a culture with clear specifications/parameters as to how things have to be done*” [low].

As *decision-making autonomy* describes employee freedom to make choices about their work processes [19, 73], employee decision authority and latitude is commonly rooted in the quality and modality of the leader-member relationships based on mutual trust between supervisors and employees [73, 117]. Decision-making autonomy was therefore operationalized as “*leadership culture*” and specified across two levels: “*trust-based*: *supervisors only control employees’ final results*” [high] and “c*ontrol-based*: *supervisors continually control employees” working progress*” [low].

In contrast to measuring work scheduling autonomy, the other two autonomy types were not explicitly operationalized to prevent the respondents drawing obvious similarities, comparisons, or perceived potential overlaps and independence issues between the autonomy dimensions and over-emphasizing their focus on these attributes [118].

Data was also collected on the three additional control organizational variables that have been found to impact employee creativity and innovation: namely, *organizational openness*, *participation in decision-making* and *formalization*.

*Organizational openness* is defined as “the degree to which individuals feel the atmosphere is conducive to the expression of individual opinions, ideas, and suggestions” [77, 119, 120]. This variable was operationalized under the label “*pressure to adjust*” and further specified into two levels: “f*reedom to be oneself*: *employees do not need to adjust themselves with regard to appearance*, *habits*, *working style*, *etc*.” [high]; and “*pressure to adjust oneself*: *employees have to adjust themselves with regard to appearance*, *habits*, *working style*, *etc*.” [low].

*Participation in decision-making* is related to an employee’s “perceived influence in joint decision making” [77, 121], and was operationalized as “*influence on company decisions*” and specified on two levels: “*democratic*: *important company decisions are taken by all employees (bottom-up)* [high]; and “*hierarchical*: *important company decisions are exclusively taken by the management team (top-down)*” [low].

*Formalization* addresses an organization’s “concern with formal rules and procedures” [81, 122, 123] and was described by “*organizational structure*” and further divided into two levels: “e*ntrepreneurial*, *flexible*: *an organization with flexible processes*” [high] and “*bureaucratic*, *standardized*: *an organization with standardized processes*” [low]. Table 2 gives an overview of the theoretical constructs and their related operationalization for the conjoint analysis.

**Table 2:** Overview of theoretical constructs and their operationalization.

Theoretical construct	Operationalization in conjoint analysis
Work scheduling autonomy (e.g., [55])	“Influence on own working conditions”
Work methods autonomy (e.g., [55])	“Experimental culture”
Decision-making autonomy (e.g., [73])	“Leadership culture”
Organizational openness (e.g., [120])	“Pressure to adjust”
Participation in decision-making (e.g., [121])	“Influence on company decisions”
Formalization (e.g., [123])	“Organizational structure”

#### Level 2. Cross-level moderators

In the post-experiment questionnaire, participants were asked about the perceived psychological climate dimensions in their organizations, for which items from Patterson, West [81] Organizational Climate Measure were selected, that broadly assessed and categorized the four different scale types: human relationships, internal processes, open systems and rational goals. Participants were presented with different types of statements and requested to; “please indicate to what extent the following statements apply to your current company.” Answers were given on a four-point Likert-type scale ranging from “1 = definitely false” to “4 = definitely true”.

*Supervisor support* was assessed using the 5 items from Patterson, West [81] and included items such as; “*supervisors here are really good at understanding peoples’ problems*”, *“supervisors show that they have confidence in those they manage”*, and *“supervisors can be relied upon to give good guidance to people”*. The internal Cronbach’s alpha reliability was .92.

*Organizational innovation* was assessed using the 6 items from Patterson, West [81] and included statements such as; “*new ideas are readily accepted here”*, “*management here are quick to spot the need to do things differently”*, “*assistance in developing new ideas is readily available*”, or “*people in this organization are always searching for new ways of looking at problems*”. The Cronbach’s alpha was .91.

*Organizational structure* was assessed using the 5 items from Patterson, West [81] and included statements such as; “*it is considered extremely important here to follow the rules*” or “*everything has to be done by the book*”. The Cronbach’s alpha was .80.

Scale reliability was furthermore tested by analyzing the split-half reliability from which the Spearman-Brown split-half coefficients and the Guttmann split-half coefficients were calculated. Overall, the results indicated and thus confirmed adequate to good reliability of the three constructs, both with regard to Spearman-Brown coefficients (supervisor support: .92, organizational innovation: .91, organizational structure: .77) and Guttman split-half coefficients (supervisor support: .89, organizational innovation: .91, organizational structure: .75). The cutoff value is commonly .60, while a value of .80 or higher indicates adequate reliability, and a value of .90 or higher indicates good reliability.

#### Additional level 2 control variables

In addition to the previously mentioned constructs, the following (level 2) variables were controlled for so as to provide a more robust interpretation of the results. First, the participants’ *age* differences were analyzed to account for any variances that may be because of age, experience or changing views and attitudes toward certain (organizational) parameters. In line with the assumption that individual preferences and perceptions change over time, participants’ *overall professional experience* and *tenure* within their current company were also tested. Both parameters were speculated to possibly have an influence on how employees perceive certain parameters within an organization.

Second, differences in the results based on the participants’ *gender* were tested. Third, different types of *company size* were controlled for, as in smaller firms, it was speculated that more responsibility and flexibility may be required from each employee, and in larger organizations, as the structures and processes may tend to be more formalized, employees have less room for discretion.

Fourth, the *industry* in which the respondents were employed was controlled for as it was speculated that autonomy might be more suitable in certain industries where flexibility, innovation and risk taking are important such as in the service sector, whereas in others such as manufacturing, consistency and control are of greater importance.

Fifth, the respondents’ *educational background* was measured as it was speculated that because more highly qualified employees generally had wider job options, they would tend to value autonomous behavior more than less qualified persons with fewer available options.

Sixth, *staff responsibility* was accounted for as employees with staff responsibility generally have greater decision powers and enhanced overall discretion in comparison to employees with no staff responsibilities.

Finally, the attribute and profile order within and across conjoint profiles were accounted for to ensure that the different arrangements of the conjoint attributes and profiles did not influence the participants’ decision-making.

## Analysis and results

The conjoint experiment yielded a total of 9,440 decisions based on 1,180 individuals from the sample that were subject to a test-retest correlation of at least .30 to ensure sufficiently high reliability [102]. The mean test-retest correlation between each individual assessment of the profile sets (i.e., 2 x 8 profile sets) was .65, only slightly below that of similar studies (e.g., .78 [108]; .78 [113]; .82 [124]), which provided assurance that there was a sufficiently high degree of judgmental consistency [109]. On level 1 the mean perceived innovative behavior score across all decisions was 4.33 (SD = 1.39). The conjoint attributes (e.g., autonomy dimensions) had means and SD of .50, reflecting the binary nature of the attributes (i.e., high vs. low). A summary of the descriptive statistics for the level 2 variables (i.e., moderators) and controls including means, standard deviations, and intercorrelations is given in Table 3.

**Table 3:** Descriptive statistics for level 2 variables including the controls (Cronbach’s alpha on the *diagonal*).

	M	SD	1.	2.	3.	4.	5.	6.	7.	8.	9.	10.	11.
1. Supervisor support	2.74	.72	.*92*	-.39**	.71**	.03*	.01	-.03	-.01	.00	.02	.01	.08*
2. Organizational structure	2.82	.59	-.39**	.*80*	*-*.43**	.01	.04	.05	.10**	-.05	-.04	-.10**	-.11**
3. Organizational innovation	2.54	.67	.71**	-.43**	.*91*	.04	.04	.01	-.04	.02	.10**	.00	.12**
4. Age	42.48	12.15	.03	.01	.04	-	.90**	.54**	-.20**	-.10*	-.02	.02	.08**
5. Years of professional experience	20.68	12.98	.01	.04	.04	.90**	-	.59**	-.16**	-.10**	-.03	-.20**	.06*
6. Years with current company / tenure	10.81	9.83	-.03	.05	.01	.54**	.59**	-	-.13**	-.18**	-.10**	-.10**	.10**
7. Gender (female)	50.3%		-.01	.10**	-.04	-.20**	-.20**	-.13**	-	.10	-.01	-.11**	-.19**
8. Company size (11–499 employees)	37.3%		.00	-.10	.02	-.10*	-.10**	-.20**	.10	-	.10	-.01	.03
9. Industry (services)	40.0%		.02	-.04	.10**	-.02	-.03	-.10**	-.01	.10	-	-.02	.01
10. Educational background (univ. degree)	46.2%		.01	-.10**	.00	.02	-.20**	-.10**	.11**	-.01	-.02	-	.17**
11. Staff responsibility (yes)	30.3%		.10*	-.11**	.12**	.08**	.06*	.10**	-.20**	.03	.01	.17**	-

Note

* p < .05.

** p < .01.

Multilevel modeling was conducted for the further analysis to account for the autocorrelation of the data. Specifically, a 2-level hierarchical linear modeling (HLM2) approach was conducted to explore the variance across different models [110]. In order to account for the nested nature of the data (i.e., decisions nested in individuals), random coefficient modeling (i.e., HLM) is well suited to deal with nested data (see also [102, 125]). The present study involved data at two levels. First, assessments of certain hypothetical contexts nested in individuals (i.e., conjoint profiles with autonomy dimensions; level 1) and, second, how higher-level variables influence these assessments (i.e., survey-based questions measuring dimensions of psychological climate; level 2). HLM is well suited for nested data, because it controls for autocorrelation and heteroskedasticity inherent in nested data [110]. HLM therefore allows to test the following three types of relationships that are also tested in this model: First, lower-level direct effects in which it is investigated whether level 1 predictors (e.g., dimensions of autonomy) have an effect on (a lower-level) outcome (e.g., perceived innovative work behavior). Second, cross-level direct effects, in which it is analyzed whether higher-level (2) predictors (e.g., psychological climate dimensions) have an effect on a lower level outcome variable (e.g. perceived innovative work behavior. And third, cross-level interaction effects in which it is analyzed whether the relationship between two lower-level variables (e.g., dimensions of autonomy with innovative work behavior) changes as a function of a higher-level variable (e.g., psychological climate dimensions).

For the model estimations, best-practice recommendations were followed as outlined in Aguinis, Gottfredson [125]. The best-practice recommendations for estimating cross-level interactions using multilevel modeling as put forth by Aguinis, Gottfredson [125] have been applied by numerous authors in the field and to date have been cited almost 270 times since their initial publication (e.g., [126, 127, 128]). The approach reflects a well-established approach in the field and was therefore also used in the analysis of this paper. Specifically, Aguinis, Gottfredson [125] recommend a sequence including four steps in the multilevel model building process.

First, an unconditional means, one-way random-effects ANOVA or *null model* is calculated in which level 1 predictors are excluded and thus only intercepts are allowed to vary across individuals. From this first step the intraclass correlation (ICC) can be calculated, which quantifies the proportion of the total variation in perceived innovative work behavior, accounted for by individual differences. Generally, a value near zero indicates that a model including level 1 variables only is suitable and that there is no need to apply multilevel modeling [125]. In cases where ICC > 0 there may be a level 2 variable (e.g., psychological climate) that explains heterogeneity of innovative behavior scores across individuals. ICC scores in multilevel studies usually range between .15 and .30 [129].

Second, a *random intercept and fixed slope model* is calculated (RIFSM) in which the level 2 equations are added. The model allows the intercepts to vary across individuals, however, slopes are not allowed to vary and the equation thus assumes that the relationship between autonomy and innovative work behavior is identical across all individuals. Third, a random intercept and random slope model is calculated (RIRSM) to test whether the third key source of variance, the variance of slopes across individuals, is different from zero (i.e., whether the relationship between autonomy and innovative work behavior varies across individuals). If such variance were nonexistent, there would be no reason for examining how certain moderators explain slope variance across individuals. Fourth, as a final step the cross-level interaction model (CLIM) is calculated to test whether a certain level 2 variable explains part of the variance in slopes across individuals (i.e., whether psychological climate moderates the relationship between autonomy and innovative work behavior across individuals).

Generally, the assumptions of multilevel modeling resemble the usual OLS regression assumptions in terms of function forms or residuals [125]. An analysis of the residuals (level 1) indicated that they were normally distributed, that there was no autocorrelation (i.e., residuals were independent from each other), and that they were homoscedastic (i.e., equal residuals across the regression line). The parameters of the model were estimated on the basis of maximum likelihood estimation. To improve the interpretation of the cross-level interaction effect, level 1 predictors were group mean-centered [125]. The correlations between level 1 variables were zero due to the orthogonal design of the experiment [98]. Table 4 provides an overview of the different model results including the coefficients, the corresponding standard errors and the significance levels.

**Table 4:** Results for multilevel modeling analysis (controls omitted).

	Model			
Level and Variable	Null	Random Intercept and Fixed Slope	Random Intercept and Random Slope	Cross-Level Interaction
**Level 1**				
Intercept	4.33*** (0.02)	4.33*** (0.02)	4.33*** (0.02)	4.33*** (0.02)
Work scheduling autonomy		0.57*** (0.02)	0.57*** (0.03)	0.57*** (0.03)
Work methods autonomy		0.60*** (0.02)	0.60*** (0.02)	0.60*** (0.02)
Decision-making autonomy		0.43*** (0.02)	0.43*** (0.02)	0.43*** (0.02)
Organizational openness		0.46*** (0.02)	0.46*** (0.02)	0.46*** (0.02)
Participation in decision-making		0.45*** (0.02)	0.45*** (0.02)	0.45*** (0.02)
Formalization		0.38*** (0.02)	0.38*** (0.02)	0.38*** (0.02)
**Level 2 (Intercept)**				
Supervisor support		0.02 (0.05)	0.05 (0.05)	0.05 (0.05)
Organizational innovation		0.16** (0.05)	0.12* (0.05)	0.13* (0.05)
Organizational structure		0.10* (0.04)	0.09* (0.05)	0.09 (0.05)
**Cross-level interactions**				
Work scheduling autonomy				
× Supervisor support				−0.06 (0.05)
× Organizational innovation				−0.02 (0.05)
× Organizational structure				0.02 (0.05)
Work methods autonomy				
× Supervisor support				0.02 (0.04)
× Organizational innovation				−0.08 (0.05)
× Organizational structure				0.00 (0.04)
Decision-making autonomy				
× Supervisor support				0.02 (0.04)
× Organizational innovation				−0.03 (0.04)
× Organizational structure				0.04 (0.03)
**Variance components**				
Intercept	0.50***	0.54***	0.60***	0.60***
Work scheduling autonomy			0.48***	0.47***
Work methods autonomy			0.32***	0.31***
Decision-making autonomy			0.20***	0.20***
Organizational openness			0.23***	0.23***
Participation in decision-making			0.30***	0.30***
Formalization			0.19***	0.19***
**Additional information**				
ICC	0.26			
−2 log likelihood FIML	31806	29062	27884	27872
Number of estimated parameters	3	12	39	48
Pseudo *R*^2^	0	0.19**	0.19**	0.19**
Model comparison χ^2^ (Degrees of Freedom)		2744.13 (9)***	1178.20 (27)***	12.10 (9)

*Note*: ICC = Intraclass correlation; FIML = full information maximum likelihood estimation; L1 = Level 1; L2 = Level2. L1 *N* = 9.440 and L2 sample size = 1.180. Values in parentheses are standard errors. Pseudo *R*^2^ values were calculated as the squared correlation between observed and predicted scores and excluded error terms [125].

* *p* < .05.

** *p* < .01.

*** *p* < .001.

### Level 1 effects

Considering the nested nature of the data, a null model for the one-way random-effects ANOVA was first calculated, from which the intraclass correlation (ICC) was calculated that quantified the proportion of the total variation in a participant’s innovative behavior that was accounted for because of employee individual differences [125]. A value of .26 indicated that there may be a level 2 variable (i.e., climate) that explained the heterogeneity of the perceived innovative behavior scores across individuals (from different organizations) with different perceived climates, indicating that multilevel modeling was appropriate [125]. Hence, this means that differences across individuals account for 26% of the variability in innovative behavior levels.

In the next steps, the random intercept fixed slope model (RIFSM; intercepts vary across individuals) and the random intercept random slope model (RIRSM; slopes vary across individuals) were calculated to test the direct effects of the level 1 autonomy dimension and the level 2 climate dimension predictors [125]. For both models all level 1 predictors were significant (*p* < .001). With regard to level 2 variables, only organization innovation and organizational structure had a significant direct effect (*p* < .05). Overall, both models were significant (RIFS model: *χ*^*2*^ = 2744.13, *p* < .001; RIRS model: *χ*^*2*^ = 1178.20, *p* < .001) and had a pseudo *R*^2^ of .19.

On level 1, the results showed significant main effects for all autonomy dimensions. Hypotheses 1a-c stated that higher levels of employee autonomy were associated with higher levels of perceived innovative work behavior, as opposed to lower levels of employee autonomy. This RIFS and RIRS model results also supported this across all measured autonomy dimensions (i.e., work scheduling autonomy (.57, *p* < .001); work methods autonomy (.60, *p* < .001), and decision making autonomy (.43, *p* < .001)) that showed significant lower-level direct effects of autonomy dimensions on perceived IWB. Significant main effects were also observed for the additional organizational control attributes (i.e., organizational openness (.46, *p* < .001); participation in decision-making (.45; *p* < .001); and formalization (.38; *p* < .001)). Of the level 2 variables only organizational innovation was found to have a significant, direct effect on the intercept (RIFSM: .16, *p* < .01; RIRSM: .12, *p* < .05).

**Fig 3:**
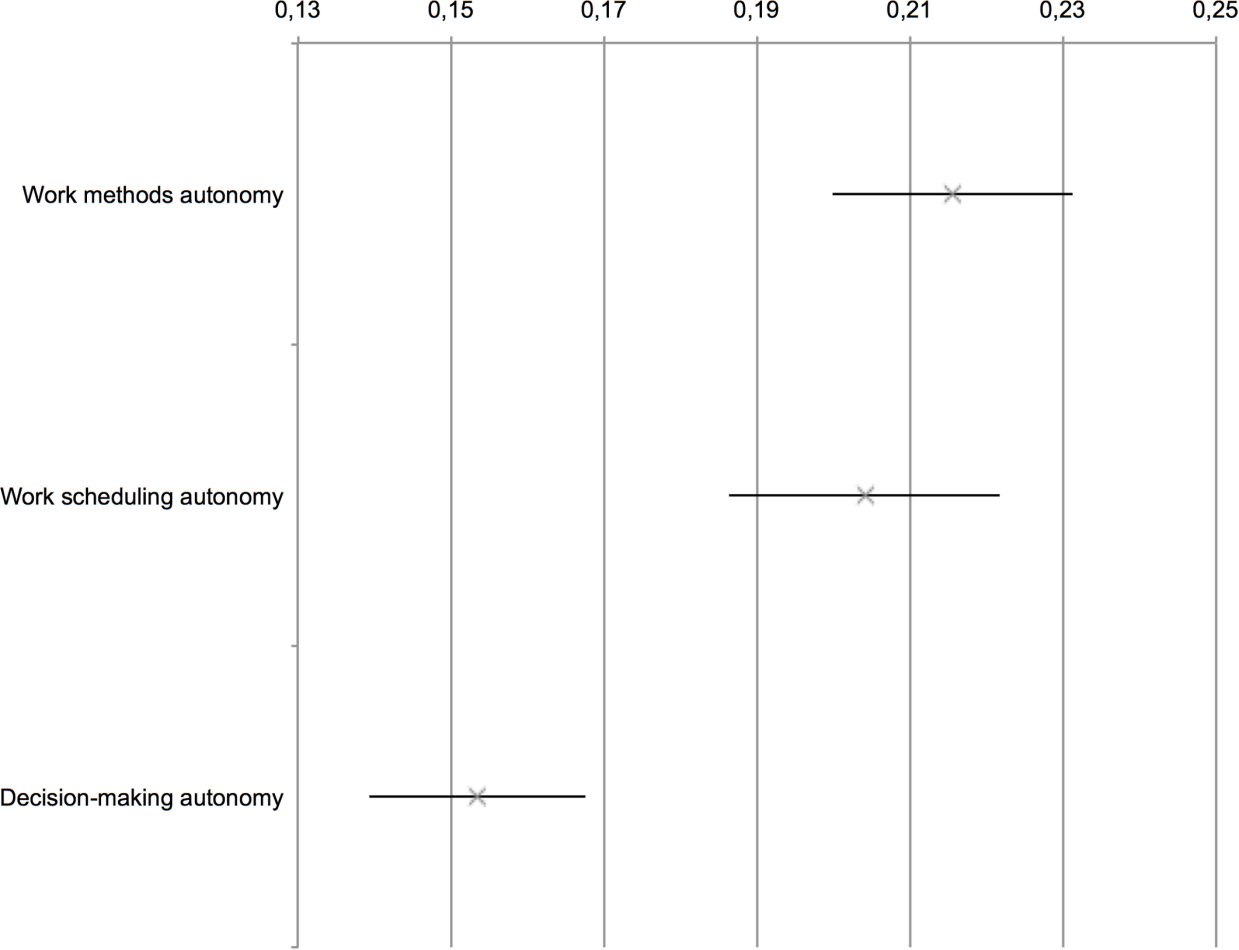
Z-standardized coefficients of autonomy attributes including 95% confidence intervals. Note: 95% confidence intervals centered around z-standardized HLM coefficients of the cross-level interactions model presented in Table 4.

Hypothesis 1d stated that work methods autonomy and decision-making autonomy had a higher and significantly more distinct effect on employee innovative behavior than work scheduling autonomy. Contrary to expectations, this Hypothesis was rejected. It was found that both work methods autonomy (95% CI [.20, .23]) and work scheduling autonomy (95% CI [.19, .22]) had an equally high influence on perceived innovative behavior, and a significantly higher effect than decision-making autonomy, (95% CI [.14, .17]). Work scheduling autonomy and work methods autonomy fell into the same confidence interval, whereas decision-making autonomy showed a significantly smaller impact on employee perceived innovative work behavior (see Fig 3). Hypothesis 1d was therefore not supported even though work methods autonomy had the strongest effect of the autonomy dimensions.

### Cross-level interaction effects between level 1 and level 2

In the final steps the recommended approach from Aguinis, Gottfredson [125] was followed and a cross-level interaction model (CLIM) calculated to analyze whether the level 2 psychological climate dimensions variable were able to explain the variance across the different organizations. This analysis evaluated how the relationship between the autonomy dimensions and employee innovative behavior was contingent on the perceived climate characteristics on level 2 [113]. Contrary to our expectation, our model was not significant (*χ*^*2*^ = 12.10, *p* > .05), with a pseudo *R*^2^ of .19.

**Fig 4:**
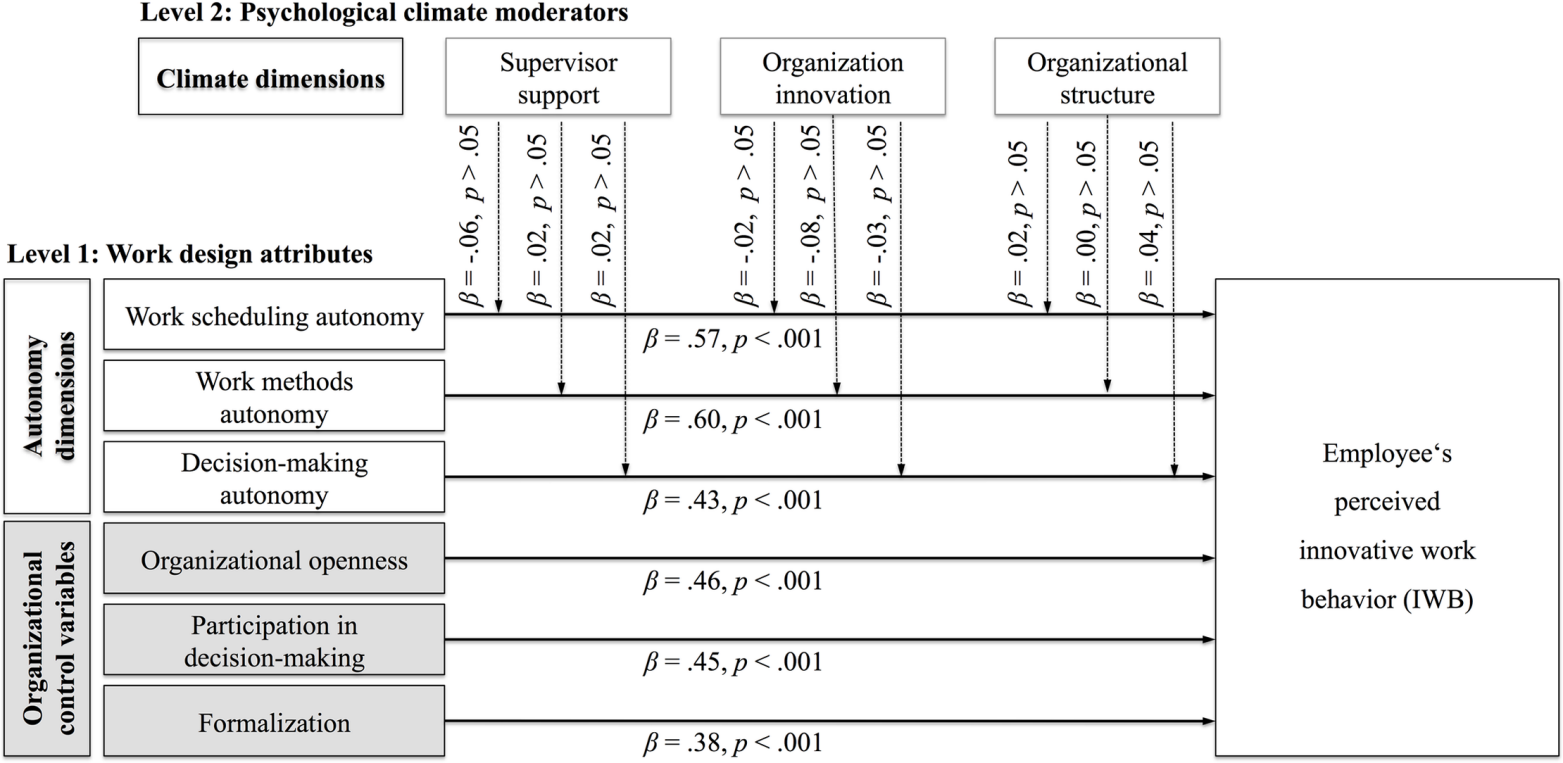
Summary of model results (without level 2 control variables).

Hypotheses 2a-c stated that supervisor support moderated the autonomy-innovative work behavior relationship. This Hypothesis was rejected, as no significant interaction effect was found. No significant interaction effects were found for the other autonomy dimensions: work scheduling autonomy, H2a (−.06, *p* > .05); work methods autonomy, H2b (.02, *p* > .05); and decision-making autonomy, H2c (.02, *p* > .05): and similar non-significant results were found for Hypotheses 3a-c, which stated that organizational innovation was a moderator in the autonomy-innovative work behavior relationship (H3a: −.02, *p* > .05; H3b: −.08, *p* > .05; H3c: −.03, *p* > .05), and Hypotheses 4a-c, which stated that organizational structure was a moderator, neither of which were supported (H4a: .02, *p* > .05; H4b: .00, *p* > .05; H4c: .04, *p* > .05). The summary of the results across the different Hypotheses is provided in Fig 4.

In addition to the analysis based on the final sample of n = 1,1180 respondents, a further analysis was conducted on the basis of the full sample of survey respondents (N = 2,550), including respondents with a test-retest correlation below .30 in the conjoint analysis (e.g., [102]) to test the robustness of the results. The summary of the results for the enlarged sample is provided in the S1 Table in the Supporting Information section. However, we decided to report the more conservative approach where we apply a similar cut-off for sample inclusion as in the extant literature (Shepherd et al., 2013). Overall, the results of the reduced sample were mainly the same in the full sample regarding Hypotheses 1–4. For Hypotheses 1a – 1c, we found that higher levels of (work scheduling, work methods, and decision-making) autonomy were also associated with higher levels of perceived innovative work behavior. Moreover, the results were even congruent with regard to effect size order, in which work methods autonomy showed the strongest effect (.32, p < .001), followed by work scheduling (.29, p < .001), and then decision-making autonomy (.22, p < .001). For Hypothesis 1d we also found that work methods autonomy and decision-making autonomy did not have a significantly larger effect on perceived innovative work behavior than work scheduling autonomy. The Hypothesis was therefore also rejected, in line with our results from the selective sample.

For Hypotheses 2–4 we also found that none of them was supported in the full sample. However, although the Hypothesis had to be rejected we found two moderating effects. First, in the full sample of respondents there was a significant interaction effect of organizational innovation on the relationship between work methods autonomy and perceived innovative work behavior (−.06, p < .05). Despite the significant (negative) effect, Hypothesis 3b still had to be rejected as it was not in line with the originally postulated direction of the Hypothesis. Second, the effect of work scheduling autonomy on perceived innovative work behavior was moderated by organizational structure (.06, p < .05), however, also in this case Hypothesis 4a had to be rejected in the full sample as the effect was not in line with the proposed direction of the Hypothesis.

Moreover, the intention of this study was to produce results that are generalizable across different contexts. Therefore, all industries were considered simultaneously in the final sample. However, we also tested the Hypotheses with regard to different industry (sub-) samples to provide additional robustness information for our analyses. Specifically, we analyzed the three largest industries in our sample, namely services (n = 472), manufacturing (n = 193), as well as retail and wholesale (n = 127). The summary of the results for the industry samples is provided in the S2–S4 Tables in the Supporting Information section of the manuscript. Overall, the industry-based results generally reflected the results from the overall sample. Hypotheses 1a – 1c were confirmed across all industries. In the services sample both the effect size and the order of magnitude of the autonomy dimensions’ effect sizes were in line with the final cross-industry sample. For the manufacturing as well as the retail and wholesale samples, effect sizes were slightly smaller and the order of magnitude of autonomy dimensions’ effect sizes differed from the overall sample.

Furthermore, Hypothesis 1d was rejected in all industry samples, which was also in line with the overall sample results. With regard to interaction effects of psychological climate dimensions on the relationship between autonomy and perceived innovative behavior (Hypotheses 2–4), the results were also mainly in line with the overall sample. None of the Hypotheses were supported and thus none of the psychological climate dimensions (i.e., supervisor support, organizational innovation, organizational structure) had a moderating effect on the analyzed relationship in the different industry samples in line with our Hypotheses. There was, however, one exception in the retail and wholesale sample in which supervisor support moderated the relationship between decision-making autonomy and perceived innovative work behavior (−.25, p < .05). Not only was the effect very small but also still led to a rejection of Hypothesis 2c as it was not in line with the direction as postulated in the Hypothesis.

Finally, an analysis was then conducted with the control variables included (see Table 5). Overall, the results also did not change considerably with regard to both the direction and significance of the effects across the different models. A few effects were noteworthy, however. Company size (1–10 employees vs. 11–499 employees) was found to have a significant, negative main effect on the intercept (RIFS model: −.20, *p* < .05; RIRS model: −.15, *p* < .05). Also, age had a significant, but small, effect in the RIRS model (−.01, *p* < .05). For the cross-level interaction effects, the profile order had a significant, negative effect with work scheduling autonomy (−.09, *p* < .05) and the attribute order had a significant, negative interaction effect with decision-making autonomy slope (−.14, *p* < .001). Interestingly, educational background (apprenticeship vs. university degree) had a significant effect on the work methods autonomy slope (−.13, *p* < .01).

**Table 5:** Results for multilevel modeling analysis including level 2 control variables.

	Model			
Level and Variable	Null	Random Intercept and Fixed Slope	Random Intercept and Random Slope	Cross-Level Interaction
**Level 1**				
Intercept	4.33*** (0.02)	4.33*** (0.02)	4.33*** (0.02)	4.33*** (0.02)
Work scheduling autonomy		0.57*** (0.03)	0.57*** (0.03)	0.57*** (0.02)
Work methods autonomy		0.60*** (0.02)	0.60*** (0.02)	0.60*** (0.02)
Decision-making autonomy		0.43*** (0.02)	0.43*** (0.02)	0.43*** (0.02)
Organizational openness		0.46*** (0.02)	0.46*** (0.02)	0.46*** (0.02)
Participation in decision-making		0.45*** (0.02)	0.45*** (0.02)	0.45*** (0.02)
Formalization		0.38*** (0.02)	0.38*** (0.02)	0.38*** (0.02)
**Level 2 (Intercept)**				
Supervisor support		0.04 (0.05)	0.05 (0.05)	0.06 (0.05)
Organizational innovation		0.15** (0.05)	0.12* (0.05)	0.13* (0.05)
Organizational structure		0.07 (0.05)	0.08 (0.05)	0.07 (0.05)
Age		0.01 (0.01)	0.01* (0.01)	0.01 (0.01)
Professional experience		0.00 (0.00)	0.00 (0.00)	0.00 (0.00)
Tenure		0.00 (0.00)	0.00 (0.00)	0.00 (0.00)
Gender		−0.05 (0.05)	−0.07 (0.05)	−0.06 (0.05)
Company size (1–10 empl. vs. 11–499 empl.)		−0.20* (0.08)	−0.15* (0.07)	−0.16* (0.08)
Industry (manufacturing vs. services)		-0.02 (0.07)	−0.03 (0.06)	−0.02 (0.06)
Educational background (apprenticeship vs. university)		0.05 (0.06)	−0.02 (0.05)	0.00 (0.06)
Staff responsibility (no resp. vs. resp.)		−0.05 (0.05)	−0.02 (0.05)	−0.03 (0.05)
Attribute order		0.08 (0.05)	0.06 (0.04)	0.07 (0.05)
Profile order		−0.03 (0.05)	−0.1 (0.04)	−0.01 (0.05)
**Cross-level interactions**				
Work scheduling autonomy				
× Supervisor support				−0.08 (0.05)
× Organizational innovation				−0.02 (0.05)
× Organizational structure				0.01 (0.05)
× Age				0.00 (0.01)
× Professional experience				0.00 (0.00)
× Tenure				0.00 (0.00)
× Gender				−0.07 (0.05)
× Company size (1–10 empl. vs. 11–499 empl.)				0.14 (0.08)
× Industry (manufacturing vs. services)				0.03 (0.07)
× Educational background (apprenticeship vs. university)				0.02 (0.06)
× Staff responsibility (no resp. vs. resp.)				0.08 (0.05)
× Attribute order				0.05 (0.05)
× Profile order				−0.09* (0.05)
Work methods autonomy				
× Supervisor support				0.01 (0.04)
× Organizational innovation				−0.07 (0.05)
× Organizational structure				0.00 (0.04)
× Age				0.00 (0.00)
× Professional experience				0.00 (0.00)
× Tenure				0.00 (0.00)
× Gender				−0.01 (0.04)
× Company size (1–10 empl. vs. 11–499 empl.)				−0.02 (0.06)
× Industry (manufacturing vs. services)				−0.03 (0.06)
× Educational background (apprenticeship vs. university)				−0.13** (0.05)
× Staff responsibility (no resp. vs. resp.)				0.07 (0.05)
× Attribute order				−0.02 (0.04)
× Profile order				0.01 (0.04)
Decision-making autonomy				
× Supervisor support				0.02 (0.04)
× Organizational innovation				−0.03 (0.04)
× Organizational structure				0.04 (0.04)
× Age				0.00 (0.00)
× Professional experience				0.00 (0.00)
× Tenure				0.00 (0.00)
× Gender				−0.03 (0.04)
× Company size (1–10 empl. vs. 11–499 empl.)				−0.02 (0.06)
× Industry (manufacturing vs. services)				−0.08 (0.05)
× Educational background (apprenticeship vs. university)				−0.05 (0.04)
× Staff responsibility (no resp. vs. resp.)				−0.01 (0.04)
× Attribute order				−0.14*** (0.04)
× Profile order				0.07 (0.04)
**Variance components**				
Intercept	0.50***	0.53***	0.59***	0.59***
Work scheduling autonomy			0.48***	0.45***
Work methods autonomy			0.32***	0.29***
Decision-making autonomy			0.20***	0.19***
Organizational openness			0.23***	0.23***
Participation in decision-making			0.30***	0.30***
Formalization			0.19***	0.19***
**Additional information**				
ICC	0.26			
−2 log likelihood FIML	31806	29042	27865	27760
Number of estimated parameters	3	27	54	108
Pseudo *R*^2^	0	0.19**	0.19**	0.20**
Model comparison χ^2^ (Degrees of Freedom)		2764 (24)***	25101 (30)***	2659 (78)***

*Note*: ICC = Intraclass correlation; FIML = Full information maximum likelihood estimation; L1 = Level 1; L2 = Level2. L1 *N* = 9.440 and L2 sample size = 1.180. Values in parentheses are standard errors. Pseudo *R*^2^ values were calculated as the squared correlation between observed and predicted scores and excluded error terms [125].

* *p* < .05.

** *p* < .01.

*** *p* < .001.

## Discussion

This article is among the first to take a context-contingent perspective in work design relationships and more specifically on the relationship between autonomy (i.e., work scheduling autonomy, work methods autonomy, and decision-making autonomy) as a key work design feature and employee perceived innovative work behavior. It therefore addresses calls for enhanced research considering the contextual features that “constrain or enhance the emergence of well-designed jobs” [29]. Furthermore, the study has also considered multiple dimensions of autonomy simultaneously, while previous studies have treated autonomy only as a unidimensional construct in the sense of work scheduling autonomy. Treating autonomy as a multi-faceted construct is important because different facets can differentially impact work outcomes such as innovative work behavior. From a methodical perspective the research approach is in line with other existing research in the field of human resource management, marketing, strategy, and organization research to study similar research questions [99, 102, 106, 107, 130]. This study is, however, among the first to investigate work design relationships in a conjoint experiment and moreover, to study the moderating effects of the organizational context.

First, contrary to the Hypotheses, organizational context was not found to moderate this investigated work design relationship. Specifically, selected psychological climate dimensions: supervisor support, organizational innovation, and organizational structure: were not found to have a moderating effect on the above relationship, such that under high/low levels, the relationship was more/less positive or the importance of the different autonomy dimensions was higher/lower. Second, however, it was found that different autonomy dimensions had direct positive effects on employee perceived innovative work behavior, and that work methods autonomy had the largest effect of the three autonomy dimensions, which was in agreement with previous work design and autonomy research (e.g., [48, 57]).

### Theoretical contributions

A key theoretical contribution of this article is that it addressed the importance of the employee autonomy–work outcome relationship and assessed whether these were always equally strong or weak or contingent on certain boundary factors and conditions. Given the assumption that employees are seeking congruence with their environments [27, 131, 132] and that the work context is undergoing rapid and frequent change [5], the results can help practitioners and researchers better understand the interrelationships between work design features and the broader organizational context.

Therefore, an analysis of the supporting and inhibitive factors in autonomy relationships indicates how organizations can adjust strategies for certain situations and effectively fine-tune work design principles and boundary conditions. Because extant work design theory and research has mostly neglected context contingent perspectives [27], this study saw autonomy as one of the most salient work design features and selected dimensions of the psychological climate as the contingency factors. The results of this study, however, confirmed the results of previous studies that have largely treated autonomy relationships in isolation independent of its moderating potential [35]. Autonomy has a significant impact on employee attitudes and work outcomes (perceived IWB) but the relationship appears to be independent from contextual boundary conditions. Contrary to expectations, the results of this study indicated that organizations do *not* need to consider the organizational context when putting together work design strategies. Similar findings were found in a study that investigated dispersed collaboration in the front-end of innovation. Specifically, the researchers have tested the moderating influence of the role of communities, as well as organizational climate on the relationship between the proficiency of dispersed collaboration and front-end innovation performance. While climate showed as well a significant direct effect on front-end innovation performance, the researchers also did not find any support for the moderating role of organizational climate in this relationship [133].

The potential reasons for these results, however, should be carefully considered in further evaluations. First, as this study only examined three selected climate dimensions, only a small part of the overall organizational context and the available climate dimensions in the affective, cognitive, and instrumental categories [28] were examined. Further, only a single work design feature within the task characteristics category (autonomy) was examined; therefore, it is likely that when the scope is broadened to include additional work design features such as task variety, feedback, job complexity or social support [35] as well as different climate dimensions [77], the results may be substantially different.

Second, one of the major underlying assumptions of this study was that employees were seeking individual correspondence with their broader work environments [132] through their work behavior, and that correspondence or, “a relationship in which the individual and the environment are corresponsive or mutually responsive” [134], supported or weakened certain relationships. Specifically, climate acts as a cross-level moderator and “shapes the relationship between work characteristics and the consequences of work design” [27]. In this study, however, this did not seem to be the case. A potential reason for this outcome might be that autonomy or its sub-dimensions do not play a role in connection with the selected climate factors. While logical theoretical connections between the autonomy–IWB relationship and the selected context dimensions that were likely to act as cross-level moderators were drawn, it seems that such congruence did not apply to the selected variables. For example, strong supervisor support was expected to positively influence the autonomy–IWB relationship through the inherent discretionary atmosphere and trust in good leader-member cooperation on which an autonomous work design can be built; however, there was no or not a strong enough connection between the climate factor and the work design attribute to trigger a cross-level interaction effect.

Moreover, a second contribution of this article is the consideration of employee autonomy as a multi-faceted construct. The results of the study clearly show that different dimensions of the construct have different results and thus clearly indicate that autonomy should not be considered as a unidimensional construct. This is insofar important, as autonomy has been one of the most salient work design features and has recently (re-)gained enhanced importance due to an increase of knowledge-based organizations in which enhanced employee autonomy has been found to be an important predictor of innovation performance [13, 26]. It is therefore important that future studies analyzing work design relationships including autonomy as a feature consider the multi-faceted nature of autonomy. In retrospective, it would therefore also be interesting to investigate which types of autonomy have been considered in previous studies and how the results would change when using other dimensions of autonomy (e.g., work methods autonomy or decision-making autonomy instead of work scheduling autonomy which has been used predominantly).

### Practical implications

From a practical perspective, this study draws organizational attention to the broader context in which companies operate and how/if organizations need to dynamically adjust their people management strategies. Given the fast changing technological environment that has impacted work processes and the occupational structure in organizations [1, 2], organizations should not treat work design changes in isolation. Instead, they need to carefully evaluate whether and how potential boundary conditions might reinforce or hamper the effect on relationships between work design features and their related outcomes. This study also confirmed that autonomy was one of the most salient work design features. Organizations need to be aware of the different autonomy dimensions and the different effects on employee attitudes and work outcomes. It is therefore crucial that based on the desired outcomes, firms have a more finely tuned understanding of the different employee autonomy dimensions so as to apply them in a more targeted way.

### Limitations and future research suggestions

Despite a rigorous methodology and a comprehensive theoretical foundation based on work design theory and contingency theory, there are several limitations. First, as this study only investigated context on the individual level through the psychological climate, the evaluations were highly dependent on individual factors, previous experience and current organizational circumstances. It was therefore not possible to draw inferences to more general work groups, departmental or organizational levels. Future studies could benefit from aggregating the individual scores to represent the climate and context on a higher level to satisfy the assumption that organizational collectives have their own climates [81].

Second, the selected (level 2) climate dimensions of supervisor support, organizational innovation and organizational structure were highly inter-correlated (see Table 3) and therefore did not represent independent climate dimensions as outlined in previous studies that have developed climate measures (e.g., [81]). More recent studies that have estimated the intercorrelations for various climate constructs seem to confirm the results of this study, indicating that the psychological climate may be represented by only the two higher dimensions of task and relational climate [77]. It might therefore be worth investigating the climate dimensions that are truly independent of each other. Related to this, the work design dimensions and climate constructs have not often been treated independently. While some studies have treated autonomy as a core task-related work design feature (e.g., [35]), others have conceived autonomy as a work context dimension (e.g., [1]). Future approaches should therefore clearly and carefully differentiate work design features and organizational context dimensions.

Third, the dependent variable and moderators only measured employee perceptions rather than the real outcomes of innovative work behavior or the prevailing organizational context conditions. Although conjoint analysis has been a proven method in similar types of research and therefore avoids many of the biases that are related to survey-based research, it remains a hypothetical (i.e., “what if”) scenario and is not able to evaluate actual outcomes of certain work designs. Employee perceptions and attitudes are a good proxy for actual conditions and work outcomes [7, 135]. This study did, however, not provide objective evidence for these outcomes or the climate conditions. Future work could therefore extend this approach and investigate the mediated relationships that measure actual work outcomes from certain work design set-ups. Likewise, such approaches could objectively measure the organizational context dimensions. Moreover, the results in this study were limited to an “expanded”/“distal” outcome of work design—innovative work behavior [5, 13]. It therefore remains unanswered as to whether similar effects apply for more proximal outcomes such as job satisfaction and motivation and how these outcomes may be related to innovative work behavior. Nevertheless, conjoint analysis remains an important method to study complex decision-making processes as it allows researchers to “assess decision-makers’ theories in use” [136]. Moreover, the method allows evaluating whether previous findings from post hoc methods can be sustained when tested using Conjoint experiments. Future studies in the field can therefore benefit from using and comparing results from conjoint analysis with those of post hoc methodologies. Finally, from the many available constructs and dimensions in each domain, this study only examined a limited set of work design features related to autonomy, and limited climate dimensions related to supervisor support, organizational innovation and organizational structure. Therefore, future studies should explore and investigate the additional relationships and more comprehensively combine the many work design features, climate dimensions and related work outcomes.

## Conclusion

Despite a comprehensive research history on work design features and their proven influence on employee attitudes and organizational work outcomes, only a few approaches have considered a context-contingent perspective and whether/how organizational boundary conditions influence such relationships. This study was among the first to take such a contextual perspective to investigate how the different employee autonomy dimensions are moderated by climate and affect innovative work behavior. A more finely-tuned understanding of these interrelationships is important so that contemporary organizations are able to cope with the rapidly changing environmental conditions and nature of work. Scholars and practitioners should be mindful of these interrelationships and rethink the seemingly known relationships between certain work design set-ups and desired employee attitudes, behaviors and work outcomes.

## Supporting information

S1 Table HLM results for full all respondents (N = 2,550). (PDF)

S2 Table HLM results for “services” (n = 472). (PDF)

S3 Table HLM results for “manufacturing” (n = 193). (PDF)

S4 Table HLM results for “retail and wholesale” (n = 127). (PDF)

S1 Appendix Survey questions incl. conjoint profile (original language and English). (PDF)

## References

[1] Wegman LA, Hoffman BJ, Carter NT, Twenge JM, Guenole N. Placing Job Characteristics in Context Cross-Temporal Meta-Analysis of Changes in Job Characteristics Since 1975. Journal of Management. 2016 10.1177/0149206316654545

[2] Colbert A, Yee N, George G. The digital workforce and the workplace of the future. Academy of Management Journal. 2016;59(3):731–739.

[3] Brynjolfsson E, McAfee A. Race against the machine: How the digital revolution is accelerating innovation, driving productivity, and irreversibly transforming employment and the economy: Brynjolfsson and McAfee; 2012.

[4] Dedrick J, Gurbaxani V, Kraemer KL. Information technology and economic performance: A critical review of the empirical evidence. ACM Computing Surveys (CSUR). 2003;35(1):1–28.

[5] Parker SK, Wall TD, Cordery JL. Future work design research and practice: Towards an elaborated model of work design. Journal of Occupational and Organizational Psychology. 2001;74(4):413–440.

[6] Johns T, Gratton L. The third wave of virtual work. Harvard Business Review. 2013;91(1):66–73.

[7] Grant AM, Parker SK. Redesigning work design theories: The rise of relational and proactive perspectives. The Academy of Management Annals. 2009;3(1):317–375.

[8] The future of jobs—Employment, skills and workforce strategy for the fourth industrial revolution [Internet]. World Economic Forum. 2016 [cited 12.08.2016]. Available from: http://reports.weforum.org/future-of-jobs-2016/.

[9] Briggs C, Makice K. Digital fluency: Building success in the digital age: SociaLens; 2012.

[10] Castells M. The information age: Economy, society, and culture—The rise of the network society. 2nd ed: John Wiley & Sons; 2011.

[11] Carnoy M, Castells M, Benner C. Labour markets and employment practices in the age of flexibility: A case study of Silicon Valley. International Labour Review. 1997;136(1):27–48.

[12] Hierarchy is overrated [Internet]. 2013 [cited 26.04.2016]. Available from: https://hbr.org/2013/11/hierarchy-is-overrated.

[13] Grant AM, Fried Y, Juillerat T. Work matters: Job design in classic and contemporary perspectives. APA handbook of industrial and organizational psychology: Building and developing and organization. 2011;1:417–453.

[14] Gottlieb J, Willmott P. The digital tipping point: McKinsey global survey results. McKinsey & Company; 2014.

[15] Digital transformation doesn’t have to leave employees behind [Internet]. 2015 [cited 01.07.2016]. Available from: https://hbr.org/2015/09/digital-transformation-doesnt-have-to-leave-employees-behind.

[16] McCord P. How netflix reinvented HR. Harvard Business Review. 2014;92(1):71–76.

[17] Mankins M, Garton E. How Spotify balances employee autonomy and accountability. Harvard Business Review 2017.

[18] Bradshaw T. Netflix subscriber growth tops estimates. Financial Times. 2017 17.10.2017.

[19] Humphrey SE, Nahrgang JD, Morgeson FP. Integrating motivational, social, and contextual work design features: a meta-analytic summary and theoretical extension of the work design literature. Journal of Applied Psychology. 2007;92(5):1332–1356. 10.1037/0021-9010.92.5.1332 17845089

[20] Fried Y, Ferris GR. The validity of the job characteristics model: A review and meta‐analysis. Personnel Psychology. 1987;40(2):287–322.

[21] Morgeson FP, Humphrey SE. Job and team design: Toward a more integrative conceptualization of work design In: Martocchio JJ, editor. Research in Personnel and Human Resources Management. 27 Bingley, UK: Emerald; 2008 p. 39–91.

[22] Hackman JR, Oldham GR. Motivation through the design of work: Test of a theory. Organizational Behavior and Human Performance. 1976;16(2):250–279.

[23] Kirkman BL, Rosen B, Tesluk PE, Gibson CB. The impact of team empowerment on virtual team performance: The moderating role of face-to-face interaction. Academy of Management Journal. 2004;47(2):175–192.

[24] Lammers J, Stoker JI, Rink F, Galinsky AD. To have control over or to be free from others? The desire for power reflects a need for autonomy. Personality and Social Psychology Bulletin. 2016;42(4):498–512. 10.1177/0146167216634064 26984014

[25] Mazmanian M, Orlikowski WJ, Yates J. The autonomy paradox: The implications of mobile email devices for knowledge professionals. Organization Science. 2013;24(5):1337–1357.

[26] Fried Y, Levi A, Laurence G. Motivation and job design in the new world of work In: Cartwright S, Cooper CL, editors. The Oxford Handbook of Personnel Psychology. Oxford, UK: Oxford University Press; 2008 p. 586–611.

[27] Morgeson FP, Dierdorff EC, Hmurovic JL. Work design in situ: Understanding the role of occupational and organizational context. Journal of Organizational Behavior. 2010;31(2‐3):351–360.

[28] Ostroff C. The effects of climate and personal influences on individual behavior and attitudes in organizations. Organizational Behavior and Human Decision Processes. 1993;56(1):56–90.

[29] Oldham GR, Hackman JR. Not what it was and not what it will be: The future of job design research. Journal of Organizational Behavior. 2010;31(2‐3):463–479.

[30] Johns G. Some unintended consequences of job design. Journal of Organizational Behavior. 2010;31(2‐3):361–369.

[31] Johns G. The essential impact of context on organizational behavior. Academy of Management Review. 2006;31(2):386–408.

[32] Parker CP, Baltes BB, Young SA, Huff JW, Altmann RA, Lacost HA, et al Relationships between psychological climate perceptions and work outcomes: A meta‐analytic review. Journal of Organizational Behavior. 2003;24(4):389–416.

[33] Lumpkin GT, Cogliser CC, Schneider DR. Understanding and measuring autonomy: An entrepreneurial orientation perspective. Entrepreneurship Theory and Practice. 2009;33(1):47–69.

[34] Hackman JR, Oldham GR. Development of the job diagnostic survey. Journal of Applied Psychology. 1975;60(2):159–170.

[35] Morgeson FP, Humphrey SE. The Work Design Questionnaire (WDQ): Developing and Validating a Comprehensive Measure for Assessing Job Design and the Nature of Work. Journal of Applied Psychology. 2006;91(6):1321–1339. 10.1037/0021-9010.91.6.1321 17100487

[36] Drazin R, Van de Ven AH. Alternative forms of fit in contingency theory. Administrative Science Quarterly. 1985;30(4):514–539.

[37] Fry LW, Smith DA. Congruence, contingency, and theory building. Academy of Management Review. 1987;12(1):117–132.

[38] Spector PE. Perceived control by employees: A meta-analysis of studies concerning autonomy and participation at work. Human Relations. 1986;39(11):1005–1016.

[39] Janz BD, Colquitt JA, Noe RA. Knowledge worker team effectiveness: The role of autonomy, interdependence, team development, and contextual support variables. Personnel Psychology. 1997;50(4):877–904.

[40] Shalley CE, Gilson LL, Blum TC. Matching creativity requirements and the work environment: Effects on satisfaction and intentions to leave. Academy of Management Journal. 2000;43(2):215–223.

[41] Lawrence PR, Lorsch JW. Organization and environment: managing differentiation and integration Boston: Harvard Business Review Press; 1986.

[42] Fiedler FE, Chemers MM. A theory of leadership effectiveness. New York: McGraw-Hill; 1967.

[43] Xiao Z, Tsui AS. When brokers may not work: The cultural contingency of social capital in Chinese high-tech firms. Administrative Science Quarterly. 2007;52(1):1–31.

[44] Yun S, Faraj S, Sims HP Jr. Contingent leadership and effectiveness of trauma resuscitation teams. Journal of Applied Psychology. 2005;90(6):1288–1296. 10.1037/0021-9010.90.6.1288 16316282

[45] Fry LW, Slocum JW. Technology, structure, and workgroup effectiveness: A test of a contingency model. Academy of Management Journal. 1984;27(2):221–246.

[46] Carnabuci G, Diószegi B. Social networks, cognitive style, and innovative performance: A contingency perspective. Academy of Management Journal. 2015;58(3):881–905.

[47] Hackman JR, Oldham GR. Work redesign. Reading, MA: Addison-Wesley; 1980.

[48] Ramamoorthy N, Flood PC, Slattery T, Sardessai R. Determinants of innovative work behaviour: Development and test of an integrated model. Creativity and Innovation Management. 2005;14(2):142–150.

[49] Langfred CW. The paradox of self-management: Individual and group autonomy in work groups. Journal of Organizational Behavior. 2000;21(5):563–585.

[50] Axtell CM, Holman DJ, Unsworth KL, Wall TD, Waterson PE, Harrington E. Shopfloor innovation: Facilitating the suggestion and implementation of ideas. Journal of Occupational and Organizational Psychology. 2000;73(3):265–285.

[51] Brock DM. Autonomy of individuals and organizations: Towards a strategy research agenda. International Journal of Business and Economics. 2003;2(1):57–73.

[52] Hartnell CA, Ou AY, Kinicki A. Organizational culture and organizational effectiveness: A meta-analytic investigation of the competing values framework's theoretical suppositions. Journal of Applied Psychology. 2011;96(4):677–694. 10.1037/a0021987 21244127

[53] Langfred CW. Too much of a good thing? Negative effects of high trust and individual autonomy in self-managing teams. Academy of Management Journal. 2004;47(3):385–399.

[54] Breaugh JA. The measurement of work autonomy. Human Relations. 1985;38(6):551–570.

[55] Breaugh JA. Further investigation of the work autonomy scales: Two studies. Journal of Business and Psychology. 1999;13(3):357–373.

[56] Yuan F, Woodman RW. Innovative behavior in the workplace: The role of performance and image outcome expectations. Academy of Management Journal. 2010;53(2):323–342.

[57] Oldham GR, Cummings A. Employee creativity: Personal and contextual factors at work. Academy of Management Journal. 1996;39(3):607–634.

[58] Van der Vegt GS, Janssen O. Joint impact of interdependence and group diversity on innovation. Journal of Management. 2003;29(5):729–751.

[59] West MA, Farr JL. Innovation at work: Psychological perspectives. Social Behaviour. 1989;4(1):15–30.

[60] Janssen O. Job demands, perceptions of effort‐reward fairness and innovative work behaviour. Journal of Occupational and Organizational Psychology. 2000;73(3):287–302.

[61] Amabile TM. The social psychology of creativity: A componential conceptualization. Journal of Personality and Social Psychology. 1983;45(2):357–376.

[62] Scott SG, Bruce RA. Determinants of innovative behavior: A path model of individual innovation in the workplace. Academy of Management Journal. 1994;37(3):580–607.

[63] Judge WQ, Fryxell GE, Dooley RS. The new task of R&D management: Creating goal-directed communities for innovation. California Management Review. 1997;39(3):72–85.

[64] Amabile TM. A model of creativity and innovation in organizations. Research in Organizational Behavior. 1988;10(1):123–167.

[65] Amabile TM, Schatzel EA, Moneta GB, Kramer SJ. Leader behaviors and the work environment for creativity: Perceived leader support. The Leadership Quarterly. 2004;15(1):5–32.

[66] Dul J, Ceylan C. The Impact of a Creativity‐supporting Work Environment on a Firm's Product Innovation Performance. Journal of Product Innovation Management. 2014;31(6):1254–1267.

[67] Volmer J, Spurk D, Niessen C. Leader–member exchange (LMX), job autonomy, and creative work involvement. The Leadership Quarterly. 2012;23(3):456–465.

[68] Wang AC, Cheng BS. When does benevolent leadership lead to creativity? The moderating role of creative role identity and job autonomy. Journal of Organizational Behavior. 2010;31(1):106–121.

[69] Schroeder RG, Van de Ven AH, Scudder GD, Polley D. The development of innovation ideas In: Van de Ven AH, Angle HL, Poole MS, editors. Research on the management of innovation: The Minnesota studies. Oxford: Oxford University Press; 1989 p. 107–134.

[70] Dorenbosch L, Engen MLv, Verhagen M. On‐the‐job innovation: The impact of job design and human resource management through production ownership. Creativity and Innovation Management. 2005;14(2):129–141.

[71] McAdam R, McClelland J. Individual and team-based idea generation within innovation management: Organisational and research agendas. European Journal of Innovation Management. 2002;5(2):86–97.

[72] Smith GF. Idea‐generation techniques: A formulary of active ingredients. The Journal of Creative Behavior. 1998;32(2):107–134.

[73] Karasek R, Brisson C, Kawakami N, Houtman I, Bongers P, Amick B. The Job Content Questionnaire (JCQ): An instrument for internationally comparative assessments of psychosocial job characteristics. Journal of Occupational Health Psychology. 1998;3(4):322–355. 9805280 10.1037//1076-8998.3.4.322

[74] De Jong JP, Den Hartog DN. How leaders influence employees' innovative behaviour. European Journal of innovation management. 2007;10(1):41–64.

[75] Zaltman G, Duncan R, Holbek J. Innovations and organizations. New York: John Wiley & Sons; 1973.

[76] Morgeson FP, Campion MA. Work design In: Borman WC, Ilgen DR, Klimoski RJ, editors. Handbook of Psychology: Industrial and Organizational Psychology. 12 Hoboken, NJ: John Wiley & Sons; 2003 p. 423–452.

[77] Benzer J, Horner M. A meta‐analytic integration and test of psychological climate dimensionality. Human Resource Management. 2015;54(3):457–482.

[78] Litwin GH, Stringer RA Jr. Motivation and organizational climate. Boston: Harvard University Press; 1968.

[79] Carr JZ, Schmidt AM, Ford JK, DeShon RP. Climate perceptions matter: A meta-analytic path analysis relating molar climate, cognitive and affective states, and individual level work outcomes. Journal of Applied Psychology. 2003;88(4):605–619. 12940402 10.1037/0021-9010.88.4.605

[80] Jones AP, James LR. Psychological climate: Dimensions and relationships of individual and aggregated work environment perceptions. Organizational Behavior and Human Performance. 1979;23(2):201–250.

[81] Patterson MG, West MA, Shackleton VJ, Dawson JF, Lawthom R, Maitlis S, et al Validating the organizational climate measure: Links to managerial practices, productivity and innovation. Journal of Organizational Behavior. 2005;26(4):379–408.

[82] Eisenberger R, Stinglhamber F, Vandenberghe C, Sucharski IL, Rhoades L. Perceived supervisor support: Contributions to perceived organizational support and employee retention. Journal of Applied Psychology. 2002;87(3):565–573. 12090614 10.1037/0021-9010.87.3.565

[83] Ilies R, Nahrgang JD, Morgeson FP. Leader-member exchange and citizenship behaviors: A meta-analysis. Journal of Applied Psychology. 2007;92(1):269–277. 10.1037/0021-9010.92.1.269 17227168

[84] Parker SK, Williams HM, Turner N. Modeling the antecedents of proactive behavior at work. Journal of Applied Psychology. 2006;91(3):636–652. 10.1037/0021-9010.91.3.636 16737360

[85] Wayne SJ, Liden RC, Kraimer ML, Graf IK. The role of human capital, motivation and supervisor sponsorship in predicting career success. Journal of Organizational Behavior. 1999;20(5):577–595.

[86] Martins E, Terblanche F. Building organisational culture that stimulates creativity and innovation. European Journal of Innovation Management. 2003;6(1):64–74.

[87] Ahmed PK. Culture and climate for innovation. European Journal of Innovation Management. 1998;1(1):30–43.

[88] Juillerat TL. Friends, not foes?: Work design and formalization in the modern work context. Journal of Organizational Behavior. 2010;31(2‐3):216–239.

[89] Pugh DS, Hickson DJ, Hinings CR, Turner C. Dimensions of organization structure. Administrative Science Quarterly. 1968;13(1):65–105.

[90] Adler PS, Borys B. Two types of bureaucracy: Enabling and coercive. Administrative science quarterly. 1996;41(1):61–89.

[91] Parker SK. Longitudinal effects of lean production on employee outcomes and the mediating role of work characteristics. Journal of Applied Psychology. 2003;88(4):620–634. 12940403 10.1037/0021-9010.88.4.620

[92] Raub S. Does bureaucracy kill individual initiative? The impact of structure on organizational citizenship behavior in the hospitality industry. International Journal of Hospitality Management. 2008;27(2):179–186.

[93] Ford CM, Gioia DA. Factors influencing creativity in the domain of managerial decision making. Journal of Management. 2000;26(4):705–732.

[94] Nayir DZ, Tamm U, Durmusoglu SS. How formalization hinders different firm innovativeness types: Opening the black box with evidence from a service industry. International Journal of Innovation and Technology Management. 2014;11(5):1–22.

[95] Cooper RG. The invisible success factors in product innovation. Journal of Product Innovation Management. 1999;16(2):115–133.

[96] Green PE, Krieger AM, Wind Y. Thirty years of conjoint analysis: Reflections and prospects. Interfaces. 2001;31(3):S56–S73.

[97] Green PE, Srinivasan V. Conjoint analysis in consumer research: Issues and outlook. Journal of Consumer Research. 1978;5(2):103–123.

[98] Patzelt H, Shepherd DA. The decision to persist with underperforming alliances: The role of trust and control. Journal of Management Studies. 2008;45(7):1217–1243.

[99] Brundin E, Patzelt H, Shepherd DA. Managers' emotional displays and employees' willingness to act entrepreneurially. Journal of Business Venturing. 2008;23(2):221–243.

[100] Baum M, Kabst R. Conjoint implications on job preferences: The moderating role of involvement. The International Journal of Human Resource Management. 2013;24(7):1393–1417.

[101] Moy JW, Lam KF. Selection criteria and the impact of personality on getting hired. Personnel Review. 2004;33(5):521–535.

[102] Shepherd DA, Patzelt H, Baron RA. “I care about nature, but…”: Disengaging values in assessing opportunities that cause harm. Academy of Management Journal. 2013;56(5):1251–1273.

[103] Donaldson SI, Grant-Vallone EJ. Understanding self-report bias in organizational behavior research. Journal of Business and Psychology. 2002;17(2):245–260.

[104] Evans DS, Leighton LS. Retrospective bias in the displaced worker surveys. Journal of Human Resources. 1995;30(2):386–396.

[105] Zacharakis AL, Meyer GD. A lack of insight: Do venture capitalists really understand their own decision process? Journal of Business Venturing. 1998;13(1):57–76.

[106] Shepherd DA, Zacharakis A. Conjoint analysis: A new methodological approach for researching the decision policies of venture capitalists. Venture Capital: An International Journal of Entrepreneurial Finance. 1999;1(3):197–217.

[107] Shepherd DA, Zacharakis A. Conjoint analysis: A window of opportunity for entrepreneurship research. Advances in entrepreneurship, firm emergence and growth. 1997;3:203–248.

[108] Shepherd DA. Venture capitalists' assessment of new venture survival. Management Science. 1999;45(5):621–632.

[109] Choi YR, Shepherd DA. Entrepreneurs’ decisions to exploit opportunities. Journal of Management. 2004;30(3):377–395.

[110] Raudenbush SW, Bryk AS, Congdon R. Hierarchical linear and nonlinear modeling. Lincolnwood, IL: Scientific Software International; 2004.

[111] Hahn GJ, Shapiro SS. A cataloging and computer program for the design and analysis of orthogonal symmetric and asymmetric fractional factorial experiments: General Electric, Research and Development Center; 1966.

[112] Hurt HT, Joseph K, Cook CD. Scales for the measurement of innovativeness. Human Communication Research. 1977;4(1):58–65.

[113] Behrens J, Ernst H, Shepherd DA. The decision to exploit an R&D project: Divergent thinking across middle and senior managers. Journal of Product Innovation Management. 2014;31(1):144–158.

[114] Breaugh JA. The work autonomy scales: Additional validity evidence. Human Relations. 1989;42(11):1033–1056.

[115] Wüllenweber K, Beimborn D, Weitzel T, König W. The impact of process standardization on business process outsourcing success. Information Systems Frontiers. 2008;10(2):211–224.

[116] Svyantek D, Bott J. Organizational culture and organizational climate measures: An integrative review. In: Thomas JC, Hersen M, editors. Comprehensive handbook of psychological assessment: Industrial and organizational assessment. 4 Hoboken, NJ: John Wiley & Sons; 2004 p. 507–524.

[117] Graen G, Novak MA, Sommerkamp P. The effects of leader—member exchange and job design on productivity and satisfaction: Testing a dual attachment model. Organizational Behavior and Human Performance. 1982;30(1):109–131.

[118] Rao VR. Applied conjoint analysis. New York, NY: Springer; 2014.

[119] O'Reilly C. Corporations, culture, and commitment: Motivation and social control in organizations. California Management Review. 1989;31(4):9–25.

[120] Flores LG, Zheng W, Rau D, Thomas CH. Organizational learning subprocess identification, construct validation, and an empirical test of cultural antecedents. Journal of Management. 2012;38(2):640–667.

[121] Connor PE. Decision-making participation patterns: The role of organizational context. Academy of Management Journal. 1992;35(1):218–232. 10117437

[122] Damanpour F. Organizational innovation: A meta-analysis of effects of determinants and moderators. Academy of Management Journal. 1991;34(3):555–590.

[123] Pierce JL, Delbecq AL. Organization structure, individual attitudes and innovation. Academy of Management Review. 1977;2(1):27–37.

[124] Patzelt H, Shepherd DA. Strategic entrepreneurship at universities: Academic entrepreneurs' assessment of policy programs. Entrepreneurship Theory and practice. 2009;33(1):319–340.

[125] Aguinis H, Gottfredson RK, Culpepper SA. Best-practice recommendations for estimating cross-level interaction effects using multilevel modeling. Journal of Management. 2013;39(6):1490–1528.

[126] Chang S, Jia L, Takeuchi R, Cai Y. Do high-commitment work systems affect creativity? A multilevel combinational approach to employee creativity. Journal of Applied Psychology. 2014;99(4):665 10.1037/a0035679 24490963

[127] Morgeson FP, Aguinis H, Waldman DA, Siegel DS. Extending corporate social responsibility research to the human resource management and organizational behavior domains: A look to the future. Personnel Psychology. 2013;66(4):805–824.

[128] Uy MA, Foo M-D, Ilies R. Perceived progress variability and entrepreneurial effort intensity: The moderating role of venture goal commitment. Journal of Business Venturing. 2015;30(3):375–389.

[129] Mathieu JE, Aguinis H, Culpepper SA, Chen G. Understanding and estimating the power to detect cross-level interaction effects in multilevel modeling. Journal of Applied Psychology. 2012;97(5):951 10.1037/a0028380 22582726

[130] Baum M, Kabst R. How to attract applicants in the Atlantic versus the Asia-Pacific region? A cross-national analysis on China, India, Germany, and Hungary. Journal of World Business. 2013;48(2):175–185.

[131] Holland JL. Making vocational choices: A theory of vocational personalities and work environments. 3rd ed. Odessa, FL, US: American Psychological Association; 1997.

[132] Dawis RV, Lofquist LH. A psychological theory of work adjustment: An individual-differences model and its applications. Minneapolis, MN: University of Minnesota Press; 1984.

[133] Bertels HM, Kleinschmidt EJ, Koen PA. Communities of practice versus organizational climate: Which one matters more to dispersed collaboration in the front end of innovation? Journal of Product Innovation Management. 2011;28(5):757–772.

[134] Lofquist LH, Dawis RV. Adjustment to work: A psychological view of man's problems in a work-oriented society. New York: Appleton-Century-Crofts, Educational Division; 1969.

[135] Ajzen I. The theory of planned behavior. Organizational Behavior and Human Decision Processes. 1991;50(2):179–211.

[136] Lohrke FT, Holloway BB, Woolley TW. Conjoint analysis in entrepreneurship research a review and research agenda. Organizational Research Methods. 2010;13(1):16–30.

