# Supplementary material for: Contextual work design and employee innovative work behavior: When does autonomy matter?
Source: PLoS One. 2018 Oct 4;13(10):e0204089. doi: 10.1371/journal.pone.0204089 (PMC6171839; doi:10.1371/journal.pone.0204089)
Supplement: S1 Table — (PDF) [file pone.0204089.s001.pdf]

**S1 Table. HLM results for full all respondents (N = 2,550).**

| | Model | | | |
|---|---|---|---|---|
| **Level and Variable** | **Null** | **Random Intercept and Fixed Slope** | **Random Intercept and Random Slope** | **Cross-Level Interaction** |
| **Level 1** | | | | |
| Intercept | 4.47*** (.02) | 4.47*** (.02) | 4.47*** (.02) | 4.47*** (.02) |
| Work scheduling autonomy | | .29*** (.01) | .29*** (.01) | .29*** (.01) |
| Work methods autonomy | | .32*** (.01) | .32*** (.01) | .32*** (.01) |
| Decision-making autonomy | | .22*** (.01) | .22*** (.01) | .22*** (.01) |
| Organizational openness | | .25*** (.01) | .25*** (.01) | .25*** (.01) |
| Participation in decision-making | | .24*** (.01) | .24*** (.01) | .24*** (.01) |
| Formalization | | .20*** (.01) | .20*** (.01) | .20*** (.01) |
| **Level 2 (Intercept)** | | | | |
| Supervisor support | | .01 (.04) | .04 (.04) | .03 (.04) |
| Organizational innovation | | .33** (.04) | .29** (.04) | .31** (.04) |
| Organizational structure | | .07* (.03) | .10** (.04) | .09* (.04) |
| **Cross-level interactions** | | | | |
| Work scheduling autonomy | | | | |
| × Supervisor support | | | | −.01 (.03) |
| × Organizational innovation | | | | −.03 (.03) |
| × Organizational structure | | | | .06* (.03) |
| Work methods autonomy | | | | |
| × Supervisor support | | | | .03 (.03) |
| × Organizational innovation | | | | −.06* (.03) |
| × Organizational structure | | | | .03 (.03) |
| Decision-making autonomy | | | | |
| × Supervisor support | | | | .02 (.02) |
| × Organizational innovation | | | | −.02 (.02) |
| × Organizational structure | | | | .03 (.02) |
| **Variance components** | | | | |
| Intercept | .74*** | .71*** | .75*** | .75*** |
| Work scheduling autonomy | | | .31*** | .31*** |
| Work methods autonomy | | | .22*** | .22*** |
| Decision-making autonomy | | | .14*** | .14*** |
| Organizational openness | | | .16*** | .16*** |
| Participation in decision-making | | | .18*** | .18*** |
| Formalization | | | .12*** | .12*** |
| **Additional information** | | | | |
| ICC | .46 | | | |
| −2 log likelihood FIML | 60381 | 57787 | 55029 | 55007 |
| Number of estimated parameters | 3 | 12 | 39 | 48 |
| Model comparison $\chi^2$ (Degrees of Freedom) | | | 2757.26 (27)*** | 2779.79(36)*** |

*Note:* ICC = Intraclass correlation; FIML = full information maximum likelihood estimation; L1 = Level 1; L2 = Level 2. L1 $N$ = 20,400 and L2 sample size = 2,550. Values in parentheses are standard errors. * $p < .05$, ** $p < .01$, *** $p < .001$.
