# Supplementary material for: Contextual work design and employee innovative work behavior: When does autonomy matter?
Source: PLoS One. 2018 Oct 4;13(10):e0204089. doi: 10.1371/journal.pone.0204089 (PMC6171839; doi:10.1371/journal.pone.0204089)
Supplement: S2 Table — (PDF) [file pone.0204089.s002.pdf]

S2 Table. HLM results for “Services” (n = 472).

	Model			
**Level and Variable**	**Null**	**Random Intercept and Fixed Slope**	**Random Intercept and Random Slope**	**Cross-Level Interaction**
**Level 1**				
Intercept	4.34*** (.04)	4.34*** (.04)	4.34*** (.04)	4.34*** (.04)
Work scheduling autonomy		.58*** (.04)	.58*** (.04)	.58*** (.04)
Work methods autonomy		.59*** (.04)	.59*** (.04)	.59*** (.04)
Decision-making autonomy		.44*** (.03)	.44*** (.03)	.44*** (.03)
Organizational openness		.47*** (.03)	.47*** (.03)	.47*** (.03)
Participation in decision-making		.43*** (.04)	.43*** (.04)	.43*** (.04)
Formalization		.39*** (.03)	.39*** (.03)	.39*** (.03)
**Level 2 (Intercept)**				
Supervisor support		−.04 (.08)	−.02 (.08)	−.01 (.08)
Organizational innovation		.28** (.09)	.26** (.09)	.26** (.09)
Organizational structure		.11 (.07)	.08 (.07)	.08 (.07)
**Cross-level interactions**				
Work scheduling autonomy				
× Supervisor support				.03 (.07)
× Organizational innovation				−.06 (.09)
× Organizational structure				−.04 (.09)
Work methods autonomy				
× Supervisor support				−.02 (.08)
× Organizational innovation				−.07 (.08)
× Organizational structure				.03 (.07)
Decision-making autonomy				
× Supervisor support				−.05 (.07)
× Organizational innovation				.10 (.07)
× Organizational structure				.03 (.05)
**Variance components**				
Intercept	.53***	.56***	.62***	.62***
Work scheduling autonomy			.42***	.42***
Work methods autonomy			.37***	.37***
Decision-making autonomy			.22***	.22***
Organizational openness			.23***	.23***
Participation in decision-making			.29***	.29***
Formalization			.17***	.17***
**Additional information**				
ICC	.27			
−2 log likelihood FIML	12808	11712	11277	11269
Number of estimated parameters	3	12	39	48
Model comparison χ^2 (Degrees of Freedom)			434.84 (27)***	7.82 (9)

Note: ICC = Intraclass correlation; FIML = full information maximum likelihood estimation; L1 = Level 1; L2 = Level 2. L1 n = 3,776 and L2 sample size = 472. Values in parentheses are standard errors. * $p < .05$, ** $p < .01$, *** $p < .001$.
