# Supplementary material for: Contextual work design and employee innovative work behavior: When does autonomy matter?
Source: PLoS One. 2018 Oct 4;13(10):e0204089. doi: 10.1371/journal.pone.0204089 (PMC6171839; doi:10.1371/journal.pone.0204089)
Supplement: S3 Table — (PDF) [file pone.0204089.s003.pdf]

S3 Table. HLM results for “Manufacturing” (n = 193).

	Model			
**Level and Variable**	**Null**	**Random Intercept and Fixed Slope**	**Random Intercept and Random Slope**	**Cross-Level Interaction**
**Level 1**				
Intercept	4.31*** (.05)	4.31*** (.05)	4.31*** (.05)	4.31*** (.05)
Work scheduling autonomy		.57*** (.05)	.57*** (.05)	.57*** (.05)
Work methods autonomy		.55*** (.05)	.55*** (.05)	.55*** (.05)
Decision-making autonomy		.36*** (.05)	.36*** (.05)	.36*** (.05)
Organizational openness		.44*** (.05)	.44*** (.05)	.44*** (.05)
Participation in decision-making		.41*** (.05)	.41*** (.05)	.41*** (.05)
Formalization		.39*** (.05)	.39*** (.05)	.39*** (.05)
**Level 2 (Intercept)**				
Supervisor support		−.01 (.10)	.03 (.11)	.02 (.11)
Organizational innovation		06 (.12)	.00 (.12)	.03 (.12)
Organizational structure		−.04 (.10)	.01 (.12)	−.02 (.12)
**Cross-level interactions**				
Work scheduling autonomy				
× Supervisor support				.05 (.10)
× Organizational innovation				.05 (.14)
× Organizational structure				.16 (.12)
Work methods autonomy				
× Supervisor support				−.11 (.09)
× Organizational innovation				−.06 (.10)
× Organizational structure				.03 (.11)
Decision-making autonomy				
× Supervisor support				.10 (.08)
× Organizational innovation				−.09 (.08)
× Organizational structure				.08 (.08)
**Variance components**				
Intercept	.41***	.45***	.51***	.51***
Work scheduling autonomy			.38***	.38***
Work methods autonomy			.30***	.30***
Decision-making autonomy			.19***	.19***
Organizational openness			.22***	.22***
Participation in decision-making			.38***	.38***
Formalization			.22***	.22***
**Additional information**				
ICC	.23			
−2 log likelihood FIML	5100	4684	4455	4445
Number of estimated parameters	3	12	39	48
Model comparison χ^2 (Degrees of Freedom)			228.45 (27)***	10.66 (36)

Note: ICC = Intraclass correlation; FIML = full information maximum likelihood estimation; L1 = Level 1; L2 = Level 2. L1 $n = 1{,}544$ and L2 sample size = 193. Values in parentheses are standard errors. * $p < .05$, ** $p < .01$, *** $p < .001$.
