# Supplementary material for: Contextual work design and employee innovative work behavior: When does autonomy matter?
Source: PLoS One. 2018 Oct 4;13(10):e0204089. doi: 10.1371/journal.pone.0204089 (PMC6171839; doi:10.1371/journal.pone.0204089)
Supplement: S4 Table — (PDF) [file pone.0204089.s004.pdf]

S4 Table. HLM results for “Retail and Wholesale” (n = 127).

	Model			
**Level and Variable**	**Null**	**Random Intercept and Fixed Slope**	**Random Intercept and Random Slope**	**Cross-Level Interaction**
**Level 1**				
Intercept	4.43*** (.07)	4.43*** (.07)	4.43*** (.07)	4.43*** (.07)
Work scheduling autonomy		.31*** (.07)	.31*** (.07)	.31*** (.07)
Work methods autonomy		.38*** (.07)	.38*** (.07)	.38*** (.07)
Decision-making autonomy		.34*** (.07)	.34*** (.07)	.34*** (.07)
Organizational openness		.26*** (.07)	.26*** (.07)	.26*** (.07)
Participation in decision-making		.37*** (.07)	.37*** (.07)	.37*** (.07)
Formalization		.22*** (.07)	.22*** (.07)	.22*** (.07)
**Level 2 (Intercept)**				
Supervisor support		.25 (.14)	.22 (.13)	.26 (.12)
Organizational innovation		−.21 (.15)	−.18 (.14)	−.20 (.13)
Organizational structure		−.03 (.14)	.01 (.13)	.01 (.11)
**Cross-level interactions**				
Work scheduling autonomy				
× Supervisor support				−.11 (.15)
× Organizational innovation				−.26 (.16)
× Organizational structure				.26 (.21)
Work methods autonomy				
× Supervisor support				.14 (.12)
× Organizational innovation				−.28 (.15)
× Organizational structure				.12 (.12)
Decision-making autonomy				
× Supervisor support				−.25* (.10)
× Organizational innovation				.08 (.11)
× Organizational structure				.00 (.12)
**Variance components**				
Intercept	.40***	.41***	.48***	.48***
Work scheduling autonomy			.86***	.86***
Work methods autonomy			.23***	.23***
Decision-making autonomy			.36***	.36***
Organizational openness			.18***	.18***
Participation in decision-making			.20***	.20***
Formalization			.15***	.15***
**Additional information**				
ICC	.24			
−2 log likelihood FIML	3309	3176	3005	2979
Number of estimated parameters	3	12	39	48
Model comparison χ^2 (Degrees of Freedom)			170.20 (27)***	25.96 (21)

Note: ICC = Intraclass correlation; FIML = full information maximum likelihood estimation; L1 = Level 1; L2 = Level 2. L1 n = 1,016 and L2 sample size = 127. Values in parentheses are standard errors. * $p < .05$, ** $p < .01$, *** $p < .001$.
