# Supplementary material for: Contextual work design and employee innovative work behavior: When does autonomy matter?
Source: PLoS One. 2018 Oct 4;13(10):e0204089. doi: 10.1371/journal.pone.0204089 (PMC6171839; doi:10.1371/journal.pone.0204089)
Supplement: S1 Appendix — (PDF) [file pone.0204089.s005.pdf]

S1 Appendix. Survey questions incl. conjoint profile (original language and English).

- Welches ist Ihr berufsqualifizierender Abschluss?
  - Abgeschlossene Berufsausbildung
  - Studium (Fachhochschule oder Universität)
  - (Noch) keine abgeschlossene Berufsausbildung
- What is your highest professional qualification?
  - Apprenticeship/vocational training
  - Academic studies
  - Professional qualification not (yet) finished
- Arbeiten Sie in Vollzeit oder Teilzeit?
  - Vollzeit
  - Teilzeit mit …%
  - Ich bin (aktuell) nicht berufstätig
- Are you currently employed full-time or part-time?
  - Full-time
  - Part-time at …%
  - I'm (currently) not employed
- Bitte geben Sie an wieviele Mitarbeiter derzeit in Ihrem Unternehmen (in Deutschland) beschäftigt sind
  - 1 bis 10
  - 11 bis 499
  - 500 bis 5,000
  - Mehr als 5,000
- Please indicate how many employees work in your company (in Germany)
  - 1 to 10 employees
  - 11 to 499 employees
  - 500 to 5,000 employees
  - More than 5,000 employees
- Bitte geben Sie ihr Geschlecht an
  - Weiblich
  - Männlich
- Please indicate your gender
  - Female
  - Male
- Bitte geben Sie Ihr Alter an
  - [… Jahre]
- Please indicate your age
  - [… years]
- In welcher Branche ist Ihr derzeitiges Unternehmen primär tätig?
  - Dienstleistungen
  - Verarbeitendes Gewerbe
  - Öffentliche Verwaltung
  - Transport, Kommunikation, Energie
  - Finanz-, Versicherungs-, und Immobilienwirtschaft
  - Baugewerbe

    - Bergbau
    - Landwirtschaft, Forstwirtschaft, Fischerei
    - Großhandel
    - Einzelhandel
    - Sonstiges, nämlich: …
    - Keine Angabe
- In which industry is your current company primarily operating?
    - Services
    - Manufacturing
    - Public administration
    - Transport, communication, energy
    - Financial, insurance, property and real estate
    - Construction
    - Mining
    - Agriculture, forestry, fishery
    - Wholesale
    - Retail
    - Other, as follows: …
    - No indication
- In welchem Unternehmensbereich bzw. in welcher Unternehmensfunktion sind Sie derzeit tätig?
    - Einkauf/Beschaffung
    - Logistik
    - Produktion
    - Forschung und Entwicklung
    - Personalwesen
    - Finanzen
    - Marketing/Vertrieb/Kommunikation
    - Informationstechnik (IT)
    - Verwaltung
    - Management
    - Sonstiges, nämlich: …
    - Keine Angabe
- In which department / function are you currently working?
    - Procurement/purchasing
    - Logistics
    - Production
    - Research and development
    - Human resources
    - Finance
    - Marketing/Sales/Communication
    - IT
    - Administration
    - Management
    - Other, as follows: …
    - No indication

- Wie lange sind Sie schon berufstätig?
    - [Jahre] [Monate]
- For how long are you already working?
    - [Years] [Months]
- Wie lange arbeiten Sie bereits in Ihrem jetzigen Unternehmen?
    - [Jahre] [Monate]
- For how long are you already working in your current company?
    - [Years] [Months]
- Tragen Sie Personalverantwortung?
    - Ja, ich trage für … Mitarbeiter Personalverantwortung
    - Nein
    - Keine Angabe
- Do you currently have staff responsibility?
    - Yes, I'm responsible for … employees
    - No
    - No indication

[Conjoint profiles, 2 x 8 profile sets]

Inwiefern trägt ein Arbeitsumfeld mit dem genannten Profil zu Ihrem innovativen Verhalten* im Job bei?

1 Trägt gar nicht dazu bei
2
3
4
5
6
7 Trägt sehr stark dazu bei

**Innovatives Verhalten umfasst die Generierung oder die Umsetzung neuer und/oder kreativer Ideen und Herangehensweisen in Ihrer Arbeit, bspw. im Hinblick auf die Verbesserung der Arbeitsqualität, Verbesserung des Kundenservice, ein besseres Produkt, ein besseres Kundenerlebnis, etc.*

Eigenschaft	Profil
**Einflussnahme auf eigene Arbeitsbedingungen**	**Selbstbestimmt:** Arbeitszeit und Arbeitsort sind im Unternehmen frei wählbar.
	**Fremdbestimmt:** Arbeitszeit und Arbeitsort werden vom Unternehmen vorgegeben.
**Experimentierkultur**	**Lernen aus Fehlern:** Eine Kultur, die es ermöglicht neue Dinge auszuprobieren.
	**Vermeiden von Fehlern:** Eine Kultur mit klaren Vorgaben, wie Dinge erledigt werden

	sollen.
**Führungskultur**	**Vertrauensbasiert:** Vorgesetzte kontrollieren lediglich das Endergebnis.
	**Kontrollbasiert:** Vorgesetzte kontrollieren stets den Arbeitsfortschritt.
**Anpassungsdruck**	**Freiheit man selbst zu sein:** Mitarbeiter müssen sich nicht anpassen bezüglich Erscheinungsbild, Gewohnheiten, Arbeitsstil, usw.
	**Druck sich anpassen zu müssen:** Mitarbeiter müssen sich anpassen bezüglich Erscheinungsbild, Gewohnheiten, Arbeitsstil, usw.
**Einflussnahme auf Unternehmensentscheidungen**	**Demokratisch:** Wichtige Unternehmensentscheidung.en werden von allen Mitarbeitern getroffen ("Bottom-Up").
	**Hierarchisch:** Wichtige Unternehmensentscheidungen werden ausschließlich vom Management getroffen ("Top-Down").
**Organisationsstruktur**	**Unternehmerisch, flexibel:** Eine Organisation mit flexiblen Prozessen.
	**Bürokratisch, standardisiert:** Eine Organisation mit standardisierten Prozessen.

Please indicate, to what extent does a working environment with the following profile contribute to your innovative behavior* at work:

1 Does not contribute at all
2
3
4
5
6
7 Contributes very much

**Innovative behavior comprises the generation or the implementation of new and/or creative ideas and approaches in your work, e.g., with regard to the improvement of the quality of your work, improvement of customer service, a better product, a better customer experience, etc.*

Characteristic	Profile
**Influence on own working conditions**	**Self-determined:** Working time and working place can be freely chosen by the employee.
	**Other-determined:** Working time and working place are determined by the company
**Experimental culture**	**Learning from mistakes:** A culture that allows employees to try out new things.

	**Prevention of failure:** A culture with clear specifications/parameters as to how things have to be done.
**Leadership culture**	**Trust-based:** Supervisors only control employees’ final results.
	**Control-based:** Supervisors continually control employees’ working progress.
**Pressure to adjust**	**Freedom to be oneself:** Employees do not need to adjust themselves with regard to appearance, habits, working style, etc.
	**Pressure to adjust oneself:** Employees have to adjust themselves with regard to appearance, habits, working style, etc.
**Influence on company decisions**	**Democratic:** Important company decisions are taken by all employees (bottom-up).
	**Hierarchical:** Important company decisions are exclusively taken by the management team (top-down).
**Organizational structure**	**Entrepreneurial, flexible:** An organization with flexible processes.
	**Bureaucratic, standardized:** An organization with standardized processes.

[Organizational climate dimensions]

[Supervisor support]

Bitte geben Sie im Folgenden an, wie sehr die jeweiligen Aussagen auf Ihr Unternehmen zutreffen bzw. nicht zutreffen:

Please indicate to what extent the following statements apply to the company you are currently working for:

- In diesem Unternehmen sind Vorgesetzte wirklich gut darin die Probleme ihrer Mitarbeiter zu verstehen.
    - 1 Stimme gar nicht zu
    - 2
    - 3
    - 4 Stimme auf jeden Fall zu
    - 5 Weiß nicht / keine Angabe
- Supervisors here are really good at understanding peoples’ problems
    - 1 Definitely false
    - 2
    - 3
    - 4 Definitely true
    - 5 Don't know / no indication
- Die Vorgesetzen zeigen, dass sie auf ihre Mitarbeitern vertrauen.
    - 1 Stimme gar nicht zu

    - 2
    - 3
    - 4 Stimme auf jeden Fall zu
    - 5 Weiß nicht / keine Angabe
- Supervisors show that they have confidence in those they manage
    - 1 Definitely false
    - 2
    - 3
    - 4 Definitely true
    - 5 Don't know / no indication
- Die Vorgesetzten sind freundlich und einfach ansprechbar.
    - 1 Stimme gar nicht zu
    - 2
    - 3
    - 4 Stimme auf jeden Fall zu
    - 5 Weiß nicht / keine Angabe
- Supervisors here are friendly and easy to approach
    - 1 Definitely false
    - 2
    - 3
    - 4 Definitely true
    - 5 Don't know / no indication
- Man kann auf die Vorgesetzten vertrauen, dass sie ihre Mitarbeiter gut führen.
    - 1 Stimme gar nicht zu
    - 2
    - 3
    - 4 Stimme auf jeden Fall zu
    - 5 Weiß nicht / keine Angabe
- Supervisors can be relied upon to give good guidance to people
    - 1 Definitely false
    - 2
    - 3
    - 4 Definitely true
    - 5 Don't know / no indication
- Die Vorgesetzten kennen und verstehen ihre Mitarbeiter sehr gut.
    - 1 Stimme gar nicht zu
    - 2
    - 3
    - 4 Stimme auf jeden Fall zu
    - 5 Weiß nicht / keine Angabe
- Supervisors show an understanding of the people who work for them
    - 1 Definitely false
    - 2
    - 3
    - 4 Definitely true
    - 5 Don't know / no indication

[Organizational structure]

Bitte geben Sie im Folgenden an, wie sehr die jeweiligen Aussagen auf Ihr Unternehmen zutreffen bzw. nicht zutreffen:

Please indicate to what extent the following statements apply to the company you are currently working for:

- Es wird in diesem Unternehmen als extrem wichtig angesehen, die Regeln zu befolgen.
  - 1 Stimme gar nicht zu
  - 2
  - 3
  - 4 Stimme auf jeden Fall zu
  - 5 Weiß nicht / keine Angabe
- It is considered extremely important here to follow the rules
  - 1 Definitely false
  - 2
  - 3
  - 4 Definitely true
  - 5 Don't know / no indication
- Mitarbeiter können formelle Prozeduren und Regeln ignorieren, wenn es ihnen dabei hilft ihre Aufgabe zu erledigen.
  - 1 Stimme gar nicht zu
  - 2
  - 3
  - 4 Stimme auf jeden Fall zu
  - 5 Weiß nicht / keine Angabe
- People can ignore formal procedures and rules if it helps get the job done
  - 1 Definitely false
  - 2
  - 3
  - 4 Definitely true
  - 5 Don't know / no indication
- Alles muss genau nach Vorschrift erledigt werden.
  - 1 Stimme gar nicht zu
  - 2
  - 3
  - 4 Stimme auf jeden Fall zu
  - 5 Weiß nicht / keine Angabe
- Everything has to be done by the book
  - 1 Definitely false
  - 2
  - 3

- 4 Definitely true
- 5 Don't know / no indication

- In diesem Unternehmen ist es nicht erfordlich Vorgehensweisen ganz genau zu folgen.
  - 1 Stimme gar nicht zu
  - 2
  - 3
  - 4 Stimme auf jeden Fall zu
  - 5 Weiß nicht / keine Angabe
- It’s not necessary to follow procedures to the letter around here
  - 1 Definitely false
  - 2
  - 3
  - 4 Definitely true
  - 5 Don't know / no indication
- In diesem Unternehmen regt sich niemand übermäßig auf, wenn Regeln gebrochen werden.
  - 1 Stimme gar nicht zu
  - 2
  - 3
  - 4 Stimme auf jeden Fall zu
  - 5 Weiß nicht / keine Angabe
- Nobody gets too upset if people break the rules around here
  - 1 Definitely false
  - 2
  - 3
  - 4 Definitely true
  - 5 Don't know / no indication

[Organizational innovation]

Bitte geben Sie im Folgenden an, wie sehr die jeweiligen Aussagen auf Ihr Unternehmen zutreffen bzw. nicht zutreffen:

Please indicate to what extent the following statements apply to the company you are currently working for:

- In diesem Unternehmen werden neue Ideen bereitwillig angenommen.
  - 1 Stimme gar nicht zu
  - 2
  - 3
  - 4 Stimme auf jeden Fall zu
  - 5 Weiß nicht / keine Angabe
- New ideas are readily accepted here
  - 1 Definitely false
  - 2

    - 3
    - 4 Definitely true
    - 5 Don't know / no indication
- Dieses Unternehmen reagiert schnell, wenn Veränderungen erforderlich sind.
    - 1 Stimme gar nicht zu
    - 2
    - 3
    - 4 Stimme auf jeden Fall zu
    - 5 Weiß nicht / keine Angabe
- This company is quick to respond when changes need to be made
    - 1 Definitely false
    - 2
    - 3
    - 4 Definitely true
    - 5 Don't know / no indication
- Das Management erkennt rasch, wenn Bedarf besteht Dinge anders zu erledigen.
    - 1 Stimme gar nicht zu
    - 2
    - 3
    - 4 Stimme auf jeden Fall zu
    - 5 Weiß nicht / keine Angabe
- Management here are quick to spot the need to do things differently
    - 1 Definitely false
    - 2
    - 3
    - 4 Definitely true
    - 5 Don't know / no indication
- Dieses Unternehmen ist sehr flexibel; es kann Vorgehensweisen rasch ändern um sich neue Gegebenheiten einzustellen und aufkommende Probleme zu lösen.
    - 1 Stimme gar nicht zu
    - 2
    - 3
    - 4 Stimme auf jeden Fall zu
    - 5 Weiß nicht / keine Angabe
- This organization is very flexible; it can quickly change procedures to meet new conditions and solve problems as they arise
    - 1 Definitely false
    - 2
    - 3
    - 4 Definitely true
    - 5 Don't know / no indication
- Unterstütztung bei der Entwicklung neuer Ideen ist stets verfügbar.
    - 1 Stimme gar nicht zu
    - 2
    - 3
    - 4 Stimme auf jeden Fall zu

- 5 Weiß nicht / keine Angabe

- Assistance in developing new ideas is readily available
  - 1 Definitely false
  - 2
  - 3
  - 4 Definitely true
  - 5 Don't know / no indication
- Mitarbeiter in diesem Unternehmen sind stets auf der Suche nach neuen Herangehensweisen für Problemstellungen.
  - 1 Stimme gar nicht zu
  - 2
  - 3
  - 4 Stimme auf jeden Fall zu
  - 5 Weiß nicht / keine Angabe
- People in this organization are always searching for new ways of looking at problems
  - 1 Definitely false
  - 2
  - 3
  - 4 Definitely true
  - 5 Don't know / no indication
